# Sustainability dimensions of diets with varying levels of food biodiversity in the NutriNet-Santé study

**DOI:** 10.1017/S1368980026102791

**Published:** 2026-05-28

**Authors:** Justine Berlivet, Bernard Srour, Benjamin Allès, Philippe Pointereau, Brigitte Langevin, Cedric Agaësse, Denis Lairon, Mathilde Touvier, Jeroen Berden, Bernadette Chimera, Giles T. Hanley-Cook, Carl Lachat, Inge Huybrechts, Julia Baudry, Emmanuelle Kesse-Guyot

**Affiliations:** 1 Université Sorbonne Paris Nord and Université Paris Cité, Inserm, INRAE, CNAM, Center of Research in Epidemiology and StatisticS (CRESS), Nutritional Epidemiology Research Team (EREN), F-39017 Bobigny, France; 2 Solagro, 75 Voie TOEC, CS 27608, F-31076 Toulouse Cedex 3, France; 3 Aix Marseille Université, Inserm, INRAE, C2VN, 13005 Marseille, France; 4 Nutrition and Metabolism Branch, International Agency for Research on Cancer, Lyon, France; 5 Department of Food Technology, Safety and Health, Faculty of Bioscience Engineer, Ghent University, Ghent, Belgium

**Keywords:** Food biodiversity, Sustainability dimensions, Diet quality, Environmental pressures, Monetary cost

## Abstract

**Objective:**

Dietary diversity is associated with health benefits. However, food biodiversity has been understudied. Therefore, we aim to evaluate the associations between dietary species richness (DSR), i.e. the number of species consumed by individuals, and dietary sustainability dimensions, including environmental pressures, diet quality and monetary cost.

**Setting:**

The study was carried out among the NutriNet-Santé cohort.

**Participants:**

29 173 volunteers were included.

**Design:**

Participants completed a 264-item FFQ. Food items were linked to the corresponding species. ANCOVA models were fitted to evaluate the associations between total, plant, animal DSR and dietary sustainability dimensions. The dietary share of organic food was computed.

**Results:**

Higher DSR was associated with lower overall environment impact, captured by the pRecipe, an indicator including greenhouse gases emissions, land occupation and cumulative energy demand (pRecipe, Q5 *v*. Q1, −8·5 %), better diet quality, reflected by the level of adherence to the French dietary guidelines (sPNNS-GS2, Q5 *v*. Q1: +44·8 %) and stable dietary cost. Higher plant DSR was related to lower environmental impacts, better diet quality and higher monetary cost. Higher animal DSR was associated with higher environmental pressures, lower diet quality and monetary cost. The amount of organic food consumed was positively associated with plant DSR and negatively associated with animal DSR.

**Conclusions:**

Overall, more biodiverse diets were related to better diet quality and lower environmental pressures. The associations seemed to be driven by plant biodiversity. Further studies are warranted to better understand the relationship between food biodiversity and sustainability dimensions across settings.

Current food systems face unprecedented environmental, nutrition and health challenges, in parallel to the world’s population estimated to reach 10 billion by 2050^(1)^. Agrifood systems are indeed major contributors to the transgression of several planetary boundaries^(2)^, with climate change, biodiversity loss and nitrogen cycle boundaries already being surpassed. Additional boundaries, such as global freshwater use, land use changes, ocean acidification and phosphorous cycle interference, are also at risk of being exceeded in the near future, pushing the planet beyond the ‘safe operating space for humanity’^(2,3)^.

In parallel, when combining fatal and nonfatal conditions, non-communicable diseases (NCD) currently account for over half of global health loss, with this proportion steadily increasing over the last 30 years^(4)^. In 2016, an estimated 40·5 million (71 %) of the 56·9 million deaths worldwide were attributable to NCD^(5)^. More specifically, in 2021, 7·22 million (95 % CI: 1·96, 10·8) deaths and 178 million (95 % CI: 49·8, 261) disability adjusted life-years were attributable to dietary risk factors^(6)^, making dietary patterns critical entry points for evidence-informed policies, programmes and interventions. Shifting towards healthy diets from sustainable agrifood systems, which balance environmental health, NCD prevention, affordability and cultural acceptability is urgently needed^(7)^. To this end, more in-depth analyses of the distinct sustainability dimensions of diets are required.

Diets with higher nutritional quality are often associated with more sustainable characteristics, even if results are dependent on the considered indicators measures and population studied^(7)^. In particular, diets with higher nutritional quality are generally associated with lower overall environmental footprint, reflected in particular by lower greenhouse gases emissions (GHGe), land occupation (LO) and lower nutrient pollution (acidification and eutrophication)^(8)^. In addition, previous research has found that dietary diversity, and especially nutritious food diversity, both between and within food groups, could be associated to better human health and disease prevention^(9)^. Likewise, almost all dietary guidelines prescribe dietary diversity as a fundamental property of a healthy diet^(10)^.

Contemporary agrifood systems are based on a more and more narrow range of plant and animal species, leading to both cultivated and wild biodiversity losses, with detrimental effects for both diet quality and environmental health, reducing the availability to a diversity of nutritious foods and contributing to the loss of ecosystem functions^(11)^. In that context, the concept of ‘food biodiversity’, defined by the FAO as the diversity of plants, animals and other organisms used for food, covering the genetic resources within species, between species and provided by ecosystems, has emerged in 2010^(12)^. Biodiverse diets potentially offer a new perspective, and their associations with sustainability dimensions of diets should be investigated.

Possible health benefits of greater food biodiversity may be explained by four conceptual hypotheses: (i) the ‘sampling effect’ whereby consuming a wide variety of edible species increases the probability of meeting nutrient requirements, simply by chance; (ii) the ‘complementary effect’, where synergistic chemical interactions between food from different species could enhance overall nutritional quality; (iii) the ‘minimising trade-offs’ effect, which reduces the risks associated with overconsumption of any single species or food group that could be detrimental to health (e.g. energy-dense, inadequate to cover nutritional requirements, or containing food contaminants or other substances that may be toxic above a certain limit) and (iv) the ‘microbiome’ effect due to a mediating influence of more diverse microbial communities, as a consequence of more biodiverse diets^(13)^.

A common measure of food biodiversity is dietary species richness (DSR), which is the absolute number of unique species consumed. Higher DSR has been associated with better nutrition and health outcomes, in particular with greater micronutrient adequacy^(14,15)^, and with lower mortality and reduced rates of gastrointestinal cancers in Europe^(13,15,16)^.

One of these studies shows that dietary GHG emissions and LO were positively associated with animal species diversity, while outcomes of plant DSR exhibit inconsistent associations^(15)^. However, these studies warrant replication, as they are based on multicentre unstandardised data. In addition, while different nutritional and environmental impacts of plant- and animal-based foods are well documented^(17,18)^, plant and animal DSR are not specifically considered in most of the existing studies.

Hence, using data from the French NutriNet-Santé cohort, the present cross-sectional study consists in a multicriteria analysis, assessing the relationships between food biodiversity evaluated using DSR and, simultaneously, three dietary sustainability dimensions, including diet quality, environmental impacts and monetary cost. The main purpose of this study is to describe the variations of meaningful and commonly used indicators according to varying levels of food biodiversity, distinguishing overall, plant and animal DSR. To our knowledge, this is the first study to examine the associations between food biodiversity and three dimensions of diet sustainability while accounting for different farming methods.

## Material and Methods

### Population

The NutriNet-Santé cohort study is an ongoing web-based cohort launched in 2009. This study is conducted in accordance with the Declaration of Helsinki, and all procedures were approved by the Institutional Review Board of the French Institute for Health and Medical Research (IRB Inserm 0000388FWA00005831) and the Commission Nationale de l’Informatique et des Libertés (CNIL 908450 and 909216). The study is also registered on ClinicalTrials.gov (NCT03335644). Electronic informed consent was obtained from all participants.

Participants are volunteers, over 18 years. At inclusion and as part of the follow-up, a large set of socio-demographic data, including sex, age, educational level, occupational status, monthly income per household unit, smoking status and physical activity level, are routinely collected using validated questionnaires^(19,20)^.

### Dietary intake data

In 2014, between June and December, among nearly 157 900 participants in the NutriNet-Santé cohort, 37 685 individuals filled in a validated 264-item FFQ assessing detailed food consumption. Participants were asked to report their consumption considering the preceding 12 months in order to prevent seasonal variations from influencing their responses.

For each item, participants were asked to estimate their consumption frequency and quantity, based on standardised photographs provided on the website, or on the value estimated by the participant. This FFQ also allowed the computation of organic food consumption in individuals’ diet, as participants were asked to answer, for each item, the following question: ‘*How often was the product of organic origin ?*’ and chose one of the following answers: never, rarely, half-of-the-time, often and always. Despite the large number of items, useful to accurately assess DSR, the questionnaire remained relatively easy to complete. Subsequently, in addition to item-level dietary data, we calculated the share of organic food in a participant’s overall diet by weighting each item’s consumption (using values 0, 0·25, 0·50, 0·75 or 1 based on the chosen modality), followed by an aggregation of all items. Methodological considerations regarding the use of the scale for assessing organic food consumption have been discussed in previous works^(21,22)^. Food items of the FFQ were aggregated into thirty-three major food groups to simplify the presentation.

### Dietary species richness

The DSR numbering procedure follows a detailed, systematic approach, derived from the FFQ items, and taking account of their diversity. Multiple sub-items could be combined into a unique FFQ item. Mixed items and sub-items are broken down into ingredients (when relevant) using generic recipes validated by nutrition experts. For each item, sub-items are weighted according to detailed data provided by the 24-h dietary records from a subset of the NutriNet-Santé cohort. The nutritional composition of each FFQ item is subsequently calculated as the weighted nutritional composition of each sub-item, with the weight reflecting the contribution of the sub-items to the item by sex, as documented in the 24-h dietary data. Species numbering is conducted similarly, referencing the FoodEx2 European database, which enumerates the corresponding species^(23)^.

Three indicators were computed: total, plant and animal DSR. Each species was categorised as animal or plant. Fungi (five species; mean (sd): 8·5 (11·4) g/d) and algae (three species: mean (sd): 2·1 (13·9) g/d) were included in the total DSR calculation, but were excluded when plant and animal DSR were considered separately.

To consider the potential inter-individual variability of homemade recipes, thresholds were applied to retain solely ingredients and species commonly used in significant quantities, as previously done in another cohort^(13)^. For each individual diet, we considered that species accounting for < 5 % (in grams) of any of the thirty-three food groups to which they pertain, were not accounted for in the total, plant or animal DSR computations. In sensitivity analyses, we also implemented the DSR indicators considering only food items, and corresponding species, consumed in amounts > 5 grams per day in an individual’s diet.

### Sustainability dimensions

#### Environmental dimension

To estimate environmental indicators, we used data derived from the DIALECTE tool, based on the Life Cycle Assessment method for sixty raw agricultural products, conducted in > 2000 French farms^(24)^. The perimeter is limited to the agricultural production stage, including the production of inputs and the supply of energy at this step, due to the lack of data concerning the downstream phases of the system for organic food. Among the farm panel, 46 % are certified as organic; thus, we were able to consider the food production method in the assessment of environmental impacts of individual diets. Allocation factors were used to estimate environmental indicators from commodities to food as consumed. Diet-related GHG emissions, energy demand and LO were obtained by multiplying the reported intake of each food item by its respective environmental indicator and summing over all food items consumed by the participant, while accounting for the production system. Missing values (*n* 32 raw agricultural products) were completed by a literature review (see online supplementary material, Supplemental Method).

In addition to these measurements, seventeen midpoints impact categories related to environmental damages were gathered into the ReCiPe index^(25)^. It has been shown that greenhouse gas emissions (GHGe, kgeqCO_2_/d), land occupation (LO, m^2^/d) and cumulative energy demand (CED, MJ/d) represent approximatively 90 % of the environmental pressures associated with diets^(26)^. Therefore, to have an aggregated measure of environmental issues associated with individuals’ diets, we used a partial version of the ReCiPe (the pRecipe), which is an aggregated score developed by the National Institute for Public Health and the Environment in the Netherlands^(25)^. It encompasses these three metrics (midpoints) that were weighted and aggregated to reflect various dimensions of the environmental impacts of individuals’ diets.

The weights associated with the different midpoints are derived from characterisation factors, normalisation and weighting procedures, based on well-known scientific mechanisms, and normative assumptions. The pRecipe formula is as follows:

*pReCiPe* = 0·0459 × *GHGe* + 0·0025 × *CED* + 0·0439 × *LO* ^(27)^

#### Nutritional dimension

To assess the nutritional dimension of diet sustainability, we considered two validated scores: the simplified Programme National Nutrition Santé − Guidelines Score 2 (sPNNS-GS2)^(28)^, and the comprehensive dietary quality index (cDQI)^(29)^. We also implemented a Food Group-based Dietary Diversity Score (FG-DDS) to evaluate the variety of food consumed by each participant.

The sPNNS-GS2 is a validated score aiming to reflect adherence to the French dietary guidelines updated in 2017 by the Haut Conseil de la Santé Publique^(30)^. In brief, sPNNS-GS2 includes thirteen dietary components regarding the main dietary recommendations, which are divided into six adequacy components and seven moderation components. Scoring and weights across components, depending on the level of evidence of the relationship with health, are based on a collective expertise. Portions were determined as usual portions in France. A penalty was deducted in case of energy intake 5 % higher than energy expenditure. The final score ranges from −∞ to 14·25^(28)^.

We also implemented the cDQI, which assesses the nutritional quality of the diet. It is composed of a vegetal subscore with eleven components and an animal subscore with six components. Each component provides 0–5 points based on increasing thresholds for beneficial components and decreasing thresholds for nonbeneficial components. Intermediate points are allocated for intermediate levels of consumption. The cDQI ranges from 0 to 85^(29)^.

Lastly, we computed the Food Group-based Dietary Diversity Score (FG-DDS), on the basis of a simple score implemented as proxy indicator of micronutrient adequacy described elsewhere^(31)^. Briefly, food items were gathered into twenty-one food groups, for which some average consumption thresholds were applied. For each food group, one point is attributed if a minimum daily amount is consumed (40 g for fruits and vegetables, 15 g for grains, legumes and cheese and 30 g for other food groups) or 0 otherwise. In this study, we adapted slightly the scoring method to our setting: we did not account for the ‘insects’ food group, and we added a ‘fat’ group, with 15 g/d as the scoring threshold.

#### Monetary cost dimension

Mean prices for the 264 food items from different places of purchase were based on data from a consumer panel of 20 000 households. Eight categories of places of purchase were distinguished, and the production method was accounted for. Data were collected in a specific questionnaire administered simultaneously to the FFQ. For food products from local markets and small farms, prices were collected by consumer association volunteers. Each participant’s daily diet monetary cost was estimated based on the quantities consumed and the corresponding item prices, taking into account the edible fraction and waste^(32)^.

### Statistical analyses

Participants were ranked and divided into quintiles, from low (Q1) to high (Q5) DSR. Participant characteristics across quintiles of total DSR are reported as means (sd) or percentages. Food consumption across quintiles of total, plant and animal DSR were also estimated. *P* values refer to two-sided tests for linear contrasts across quintiles for continuous variables or the Mantel–Haenszel *χ* ^2^ trend for categorical variables.

Food group consumption levels across total DSR quintiles, adjusted for age, sex and alcohol-free energy intake, were calculated, using the 5 % cut-off presented in the section above. Thereafter, food consumption across plant DSR quintiles (based on the 5 % cut-off) was also estimated, after adjustment for age, sex, alcohol-free energy intake and animal DSR. The same calculation was made for food consumption across animal DSR quintiles, with adjustment for age, sex, alcohol-free energy intake and plant DSR. ANCOVA models were performed to assess the associations between DSR and sustainability indicators, providing adjusted means (95 % CI). The main models were implemented using the 5 % cut-off. A first model (Model 1) was adjusted for alcohol-free energy intake. A second model (Model 2) was further adjusted for age and sex (main model). A third model (Model 3) was Model 2 further adjusted for the amount of plant protein (g/d) in the diet, as a proxy of the share of plant-based food in the diet.

For the main model, the sustainability metrics (reflecting the nutrition, environment and cost dimensions) were considered as dependent variables and DSR (continuous or quintiles), sex (binary), age (continuous) and energy intake without alcohol (continuous) as independent variables.

The analyses were then conducted separately for plant and animal DSR. The same analyses were carried out for these two components of DSR. Model 4 assessed the associations between plant DSR and sustainability indicators, adjusted for Model 2 covariates and animal DSR, as a continuous variable, and Model 6 explored the associations between animal DSR and sustainability indicators, adjusted for Model 2 covariates and plant DSR, as a continuous variable. In both cases, we also computed analyses further adjusted for the share of organic food in the diet (Model 5 and Model 7). For each model, the estimates and their corresponding confidence intervals for each quintile are presented graphically. All the analyses were repeated using the 5 g/d cut-off.

Two-sided tests were used, and a *P* value < 0·05 was considered significant. Data management and statistical analyses were performed using SAS® (namely SAS® GLM procedure) (version 9.4; SAS Institute, Inc.).

## Results

**Figure 1.**
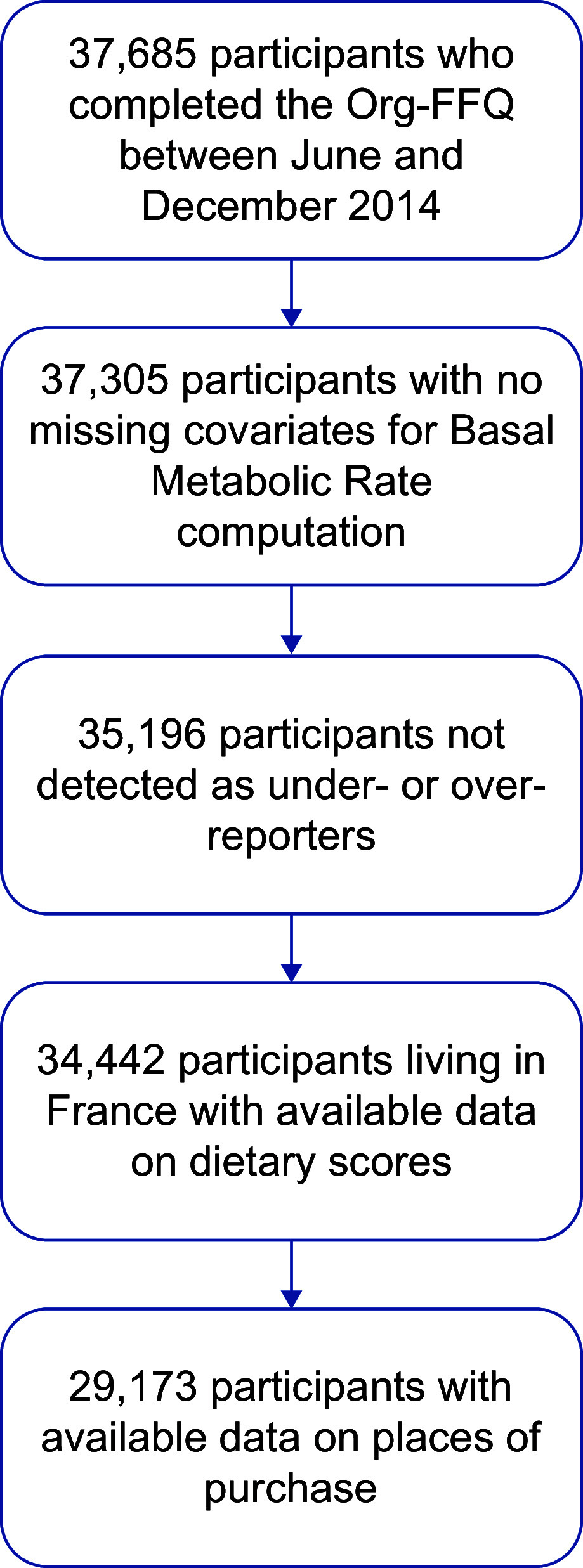
Flow chart of the study sample.

DSR was calculated for 34 442 individuals (participants filling in the FFQ in 2014 and having available required data), among whom 29 173 individuals completed the questionnaire inquiring places of purchases data (final sample, Figure 1).

The population comprised 21 807 (75 %) females and the mean (sd) age was 53·6 (14·0) years. In this sample, the number of plant species consumed varied between 2 and 78 and animal species from 0 to 33. The mean (sd) total, plant and animal DSR were 69·6 (10·1), 47·4 (8·5), 21·9 (4·0), respectively.

The characteristics of the study sample across the total DSR quintiles are shown in Table 1. The mean (sd) age decreased across DSR quintiles, from 54·3 (14·4) years old in Q1 to 51·7 (13·7) years old in Q5. The proportion of intermediate and intellectual professions increased across quintiles, whereas the share of retired participants, employees and manual workers declined. Current smokers were more represented in the lowest quintiles of overall DSR. Mean (sd) alcohol-free energy intake increased with total DSR (from 1800 (619) in Q1 to 2048 (619) kcal/d in Q5) (Table 1).

**Table 1.** Characteristics of the study sample across total DSR quintiles (cut-off 5 %)^*^ Table 1 long description.

	Q1 (*n* 5758) %	Q2 (*n* 5759) %	Q3 (*n* 6418) %	Q4 (*n* 5620) %	Q5 (*n* 5618) %	*P* ^‡^
Total DSR	< 62	63–68	69–72	73–77	> 78	
Female, %	75·22	76·23	74·48	73·22	74·60	0·01
Male, %	24·78	23·77	25·52	26·78	25·40	
Age, years						
Mean	54·3	54·2	54·0	53·6	51·7	< 0·0001
sd	14·4	14·2	13·9	13·5	13·7	
Occupational status, %						
Unemployed (others)	5·85	5·12	4·43	4·61	4·72	< 0·0001
Unemployed	5·09	3·87	3·43	3·58	4·38	
Student	2·22	1·86	1·79	1·69	2·10	
Retired	40·40	39·26	38·80	37·03	30·70	
Farmer, self-employed	2·05	1·63	1·77	1·32	0·20	
Intellectual profession	15·27	18·93	21·13	23·83	26·09	
Employee	16·74	13·75	12·84	11·51	11·93	
Manual worker	1·23	1·08	0·84	0·80	0·78	
Intermediate profession	11·15	14·50	14·97	15·64	17·34	
Educational level, %						
< High school	29·18	22·96	20·89	18·51	14·81	< 0·0001
High school	17·07	15·80	14·44	13·27	12·58	
> High school	53·75	61·24	64·66	68·22	72·61	
Monthly income per household unit (€), %						
< 1200	15·77	11·36	10·55	10·1	10·27	0·23
1200–2300	41·51	39·54	38·35	38·15	39·28	
2300–3700	24·56	30·23	30·88	31·66	31·91	
> 3700	10·40	12·42	14·68	14·68	13·71	
Unwilling to answer	7·76	6·46	5·55	5·41	5·07	
Smoking status, %						
Non smoker	46·09	48·25	48·83	49·50	51·33	0·01
Former smoker	41·30	40·08	40·81	41·17	38·95	
Current smoker	12·61	11·67	10·36	9·32	9·72	
Physical activity level (MET-min/week), %						
Missing data^†^	13·23	11·70	10·03	10·21	8·72	< 0·0001
High (> 1500)	34·65	33·36	33·52	32·28	34·39	
Moderate (600–1500)	31·64	35·32	36·57	38·90	39·59	
Low (< 600)	20·48	19·62	19·88	18·61	17·30	
Energy intake without alcohol						
Mean	1800	1901	1965	1994	2048	< 0·0001
sd	619	598	602	601	619	

Q,: quintile; DSR, dietary species richness; MET, metabolic equivalent of task.

* Species accounting for < 5 % (in grams) of any of the thirty-three food groups to which they pertain were not accounted for in the total, plant or animal DSR computations, respectively.

† Missing data as some questions are optional.

‡ *P*-values refer to two-sided tests for linear contrasts across quintiles for continuous variables or the Mantel–Haenszel *χ* ^2^ trend for categorical variables.

**Figure 2.**
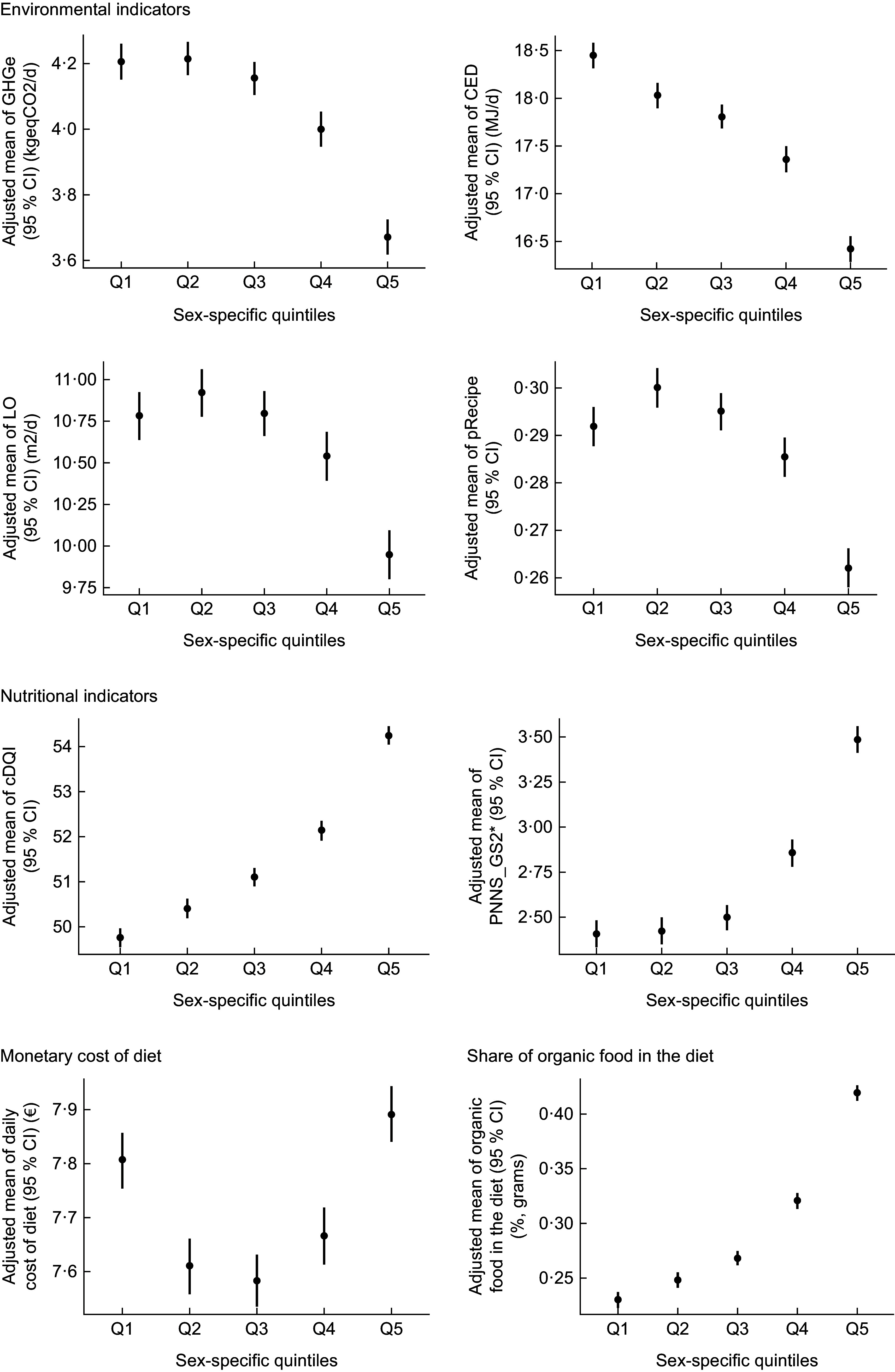
Sustainability indicators and total DSR (cut-off 5 %) assessed by quintiles or as a continuous variable. DSR, dietary species richness.

Food group intakes across DSR indexes are presented in online supplementary material, Supplemental Tables S1–S3. Our main findings, from Model 2, are presented in Figure 2, online supplementary material, Supplemental Table S4. In Model 2, the indicators of environmental pressures, namely GHGe, LO, CED and the pRecipe decreased across total DSR quintiles (GHGe: Q5 *v*. Q1: 3·67 (3·62; 3·73) *v*. 4·21 (4·15; 4·26), i.e. −12·8 %; LO: Q5 *v*. Q1: 9·95 (9·80; 10·10) *v*. 10·78 (10·64; 10·93), i.e. −7·7 %; CED: Q5 *v*. Q1: 16·42 (16·29; 16·56) *v*. 18·46 (18·32; 18·59), i.e. −11·1 %; pRecipe: Q5 *v*. Q1: 0·65 (0·64; 0·66) *v*. 0·71 (0·70; 0·72), i.e. −8·5 %). The overall diet quality increased (cDQI, Q5 *v*. Q1: 54·23 (54·00; 54·45) *v*. 49·76 (49·54; 49·98), i.e. +9·0 %), and adherence to the French food-based dietary guidelines was greater in the highest quintiles (sPNNS-GS2, Q5 *v*. Q1: 3·49 (3·41; 3·56) *v*. 2·41 (2·33; 2·48), i.e. +44·8 %). The same was true for FG-DDS (Q5 *v*. Q1: 9·52 (9·47; 9·57) *v*. 8·44 (8·39; 8·48), i.e. +12·8 %). FG-DDS was highly correlated with total DSR when considered as a continuous variable (*ρ* = 0·86). Furthermore, the share of organic food in the diet increased between the lowest and the highest quintile of total DSR (Q5 *v*. Q1: 0·42 (0·41; 0·43) *v*. 0·23 (0·22; 0·24), i.e. +82·6 %). The monetary cost of diets was not differentiated by total DSR (Figure 2, online supplementary material, Supplemental Table S4). For information, we also presented the results of the model only adjusted for alcohol-free energy intake (online supplementary material, Supplemental Table S4).

When adjusting for the quantity of plant protein in the diet, GHGe, LO and CED slightly decreased between Q1 and Q5 (GHGe: Q5 *v*. Q1: 3·91 (3·86; 3·95) *v*. 4·15 (4·10; 4·20), i.e. −5·8 %; LO: Q5 *v*. Q1: 10·49 (10·35; 10·62) *v*. 10·66 (10·52; 10·79), i.e. −1·6 %; CED: Q5 *v*. Q1: 16·96 (16·84; 17·08) *v*. 18·33 (18·21; 18·49), i.e. −7·5 %, as did the pRecipe (Q1: 0·70 (0·70; 0·71) *v*. Q5: 0·68 (0·67; 0·69), i.e. −2·9 %). The positive associations between either cDQI or sPNNS-GS2 and DSR quintiles were attenuated (cDQI: Q5 *v*. Q1: 53·56 (53·35; 53·78) *v*. 49·91 (49·70; 50·12), i.e. +7·3 % and sPNNS-GS2: Q5 *v*. Q1 3·15 (3·08; 3·22) *v*. 2·49 (2·42; 2·55), i.e. +26·5 %, respectively). The share of organic food in the diet increased between Q1 and Q5 (Q5 *v*. Q1: 0·39 (0·39; 0·40) *v*. 0·24 (0·23; 0·24), i.e. +62·5 %). The monetary cost of the diet remained stable between the extreme quintiles (Q5 *v*. Q1: 7·79 (7·73; 7·84) *v*. 7·83 (7·78; 7·88), i.e. −0·5 %) (online supplementary material, Supplemental Table S5).

**Figure 3.**
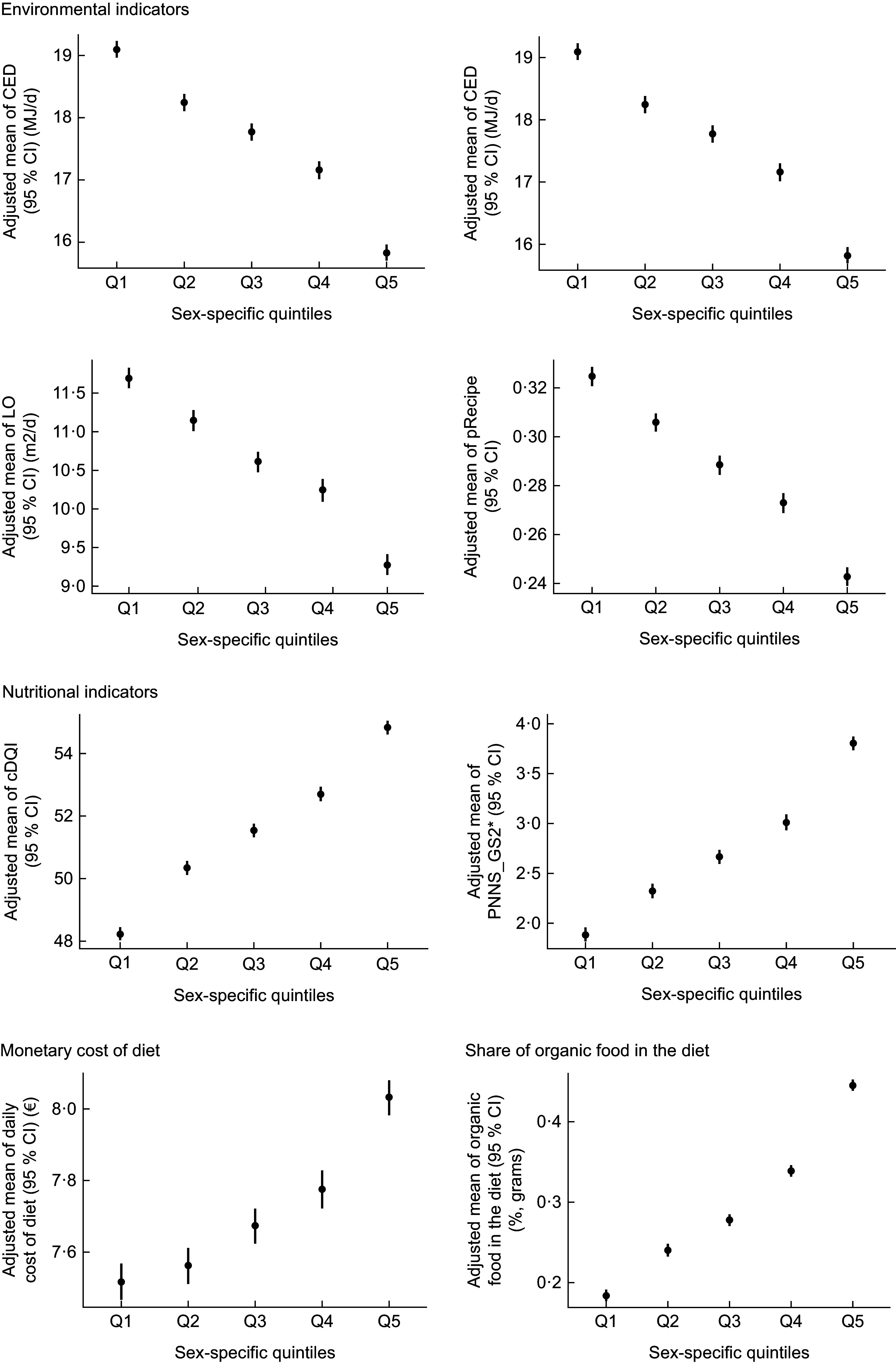
Sustainability indicators and plant DSR (cut-off 5 %) assessed by quintiles or as a continuous variable. DSR, dietary species richness.

After adjustment for alcohol-free energy intake, age, sex and animal DSR, plant DSR was inversely associated with the pRecipe (Q5 *v*. Q1: 0·60 (0·59; 0·61) *v*. 0·77 (0·76; 0·78), i.e. −25 %), positively associated with cDQI (Q5 *v*. Q1: 54·82 (54·61; 55·03) *v*. 48·25 (48·04; 48·46), i.e. +13·6 %) and sPNNS-GS2 (Q5 *v*. Q1: 3·80 (3·73; 3·87) *v*. 1·88 (1·81; 1·95), i.e. +102·1 %), and the FG-DDS increased by 11·3 % between Q1 and Q5 (Q5 *v*. Q1: 9·56 (9·52; 9·61) *v*. 8·36 (8·32; 8·41)) The share of organic food in the diet also increased (Q5 *v*. Q1: 0·45 (0·44; 0·45) *v*. 0·18 (0·18; 0·19), i.e. +150·0 %) with greater plant DSR. Finally, increasing the number of plant species consumed was associated with a slight additional cost (Q5 *v*. Q1: 8·03 (7·98; 8·08) *v*. 7·51 (7·46; 7·56), i.e. +6·9 %) (Figure 3, online supplementary material, Supplemental Table S6). When adjusting further for the amount of organic food in the diet, the associations remained consistent but slightly weakened. In summary, the pRecipe declined with plant DSR (Q5 *v*. Q1: 0·63 (0·62; 0·63) *v*. 0·75 (0·75; 0·76), i.e. −16 %), while cDQI and sPNNS-GS2 increased with plant DSR between Q1 and Q5 (cDQI: 53·54 (53·34; 53·73) *v*. 49·21 (49·00; 49·42), i.e. +8·8 % and sPNNS-GS2: Q5 *v*. Q1: 3·37 (3·30; 3·44) *v*. 2·20 (2·13; 2·37), i.e. +53·2 %) and FG-DDS increased of 11·5 % between Q1 and Q5 (Q5 *v*. Q1: 9·43 (9·39; 9·48) *v*. 8·46 (8·41; 8·51)). The monetary cost of the diet remained stable across quintiles of plant DSR (online supplementary material, Supplemental Figure S1, Table S6).

**Figure 4.**
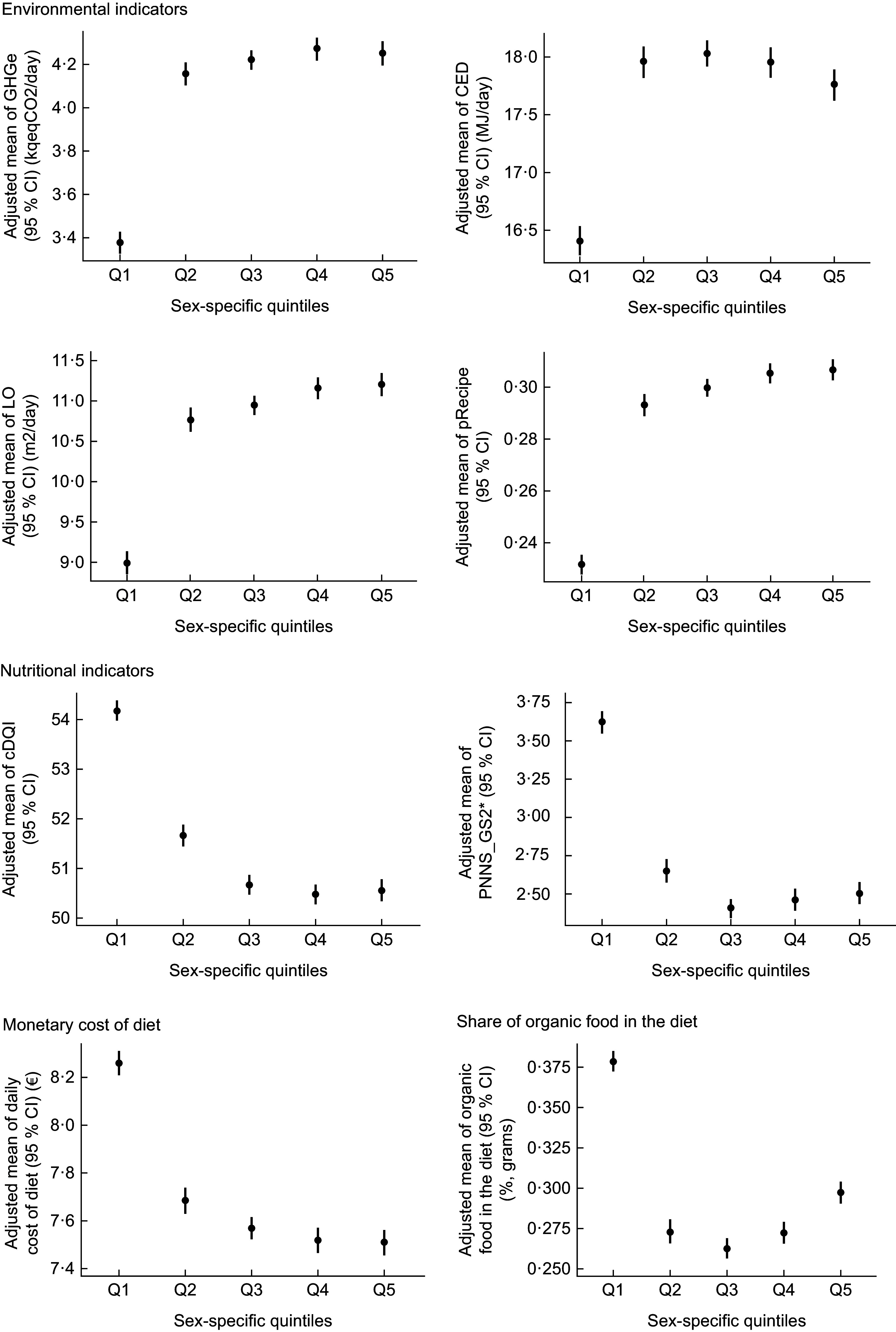
Sustainability indicators and animal DSR (cut-off 5 %) assessed by quintiles or as a continuous variable. DSR, dietary species richness.

For the associations regarding animal DSR, after adjustment for plant DSR, the pRecipe increased across quintiles (Q5 *v*. Q1: 0·73 (0·72; 0·74) *v*. 0·59 (0·58; 0·60), i.e. +23·7 %), while cDQI and sPNNS-GS2 decreased (cDQI: Q5 *v*. Q1: 50·57 (50·34; 50·79) *v*. 54·17 (53·96; 54·38), i.e. −6·6 % and sPNNS-GS2: Q5 *v*. Q1: 2·51 (2·43; 2·59) *v*. 3·62 (3·55; 3·70), i.e. −30·7 %, respectively). FG-DDS remained stable between Q1 and Q5 (Q5 *v*. Q1: 8·90 (8·85; 8·95) *v*. 9·08 (9·03; 9·12), i.e. −2·0 %), and the monetary cost of the diet slightly declined (Q5 *v*. Q1: 7·51 (7·46; 7·57) *v*. 8·26 (8·21; 8·31), i.e. −9·1 %) (Figure 4, online supplementary material, Supplemental Table S7). After adjustment for the amount of organic food in the diet, the associations were slightly attenuated. In brief, pRecipe increased by 21·7 % between Q1 and Q5 (Q5 *v*. Q1: 0·73 (0·72; 0·74) *v*. 0·60 (0·59; 0·61)), and the overall quality of diet still decreased (cDQI: Q5 *v*. Q1: 50·55 (50·34; 50·77) *v*. 53·48 (53·27; 53·69), i.e. −5·5 %), as did the level of adherence to French dietary guidelines (Q5 *v*. Q1: 2·50 (2·43; 2·58) *v*. 3·38 (3·31; 3·45), i.e. −26·0 %). FG-DDS still remained stable (Q1 *v*. Q5: 8·90 (8·85; 8·95) *v*. 9·01 (8·96; 9·06), i.e. −1·2 %). The monetary cost of diet declined by 6·9 % between the extreme quintiles (Q1 *v*. Q5: 7·51 (7·45; 7·56) *v*. 8·07 (8·02; 8·12)) (online supplementary material, Supplemental Figure S2, Table S7).

Participants characteristics using the 5 grams cut-off to compute overall DSR are presented in online supplementary material, Supplemental Table S8. Using this cut-off value did not change the findings substantially. However, in model 2, adjusted for alcohol-free energy intake, sex and age, we observed that CED remained stable (Q5 *v*. Q1: 17·61 (17·46; 17·76) *v*. 17·22 (17·07; 17·37), i.e. −2·3 %). Overall diet quality increased (cDQI, Q5 *v*. Q1: (58·29 (58·07; 58·50) *v*. 44·30 (44·08; 44·51), i.e. +31·8 %). The adherence to French dietary guidelines also drastically increased with increasing DSR (sPNNS-GS2, Q5 *v*. Q1: 4·60 (4·53; 4·68) *v*. 0·65 (0·57; 0·73), i.e. +607·6 %), and FG-DDS also increased (Q5 *v*. Q1: 10·80 (10·76; 10·85) *v*. 6·77 (6·72; 6·81), i.e. +59·5 %). The increase in the share of organic food in individual diets was noticeable (Q5 *v*. Q1: 0·48 (0·47; 0·49) *v*. 0·14 (0·13; 0·15), i.e. +242·9 %), and the monetary cost of diets increased by 38·8 % between Q1 and Q5 (Q5 *v*. Q1: 9·23 (9·18; 9·28) *v*. 6·65 (6·60; 6·71)) (online supplementary material, Supplemental Table S9). Adjusting for the amount consumed of plant-based protein did not change the findings (online supplementary material, Supplemental Table S10). Focusing on plant DSR or animal DSR also led to the same conclusions as above, except for cDQI and the cost of diet which increased between animal DSR quintiles (Q5 *v*. Q1: 53·98 (53·79; 54·18) *v*. 49·05 (48·85; 49·25), i.e. +10·1 % and Q5 *v*. Q1: 8·46 (8·42; 8·51) *v*. 7·32 (7·27; 7·37), i.e. +29·4 %, respectively). Overall, the associations remained consistent after adjustment for the amount of organic food in the diet, except for CED which increased slightly across plant DSR quintiles (online supplementary material, Supplemental Tables S11–S12).

## Discussion

The present cross-sectional study aims to evaluate, using a multicriteria approach, the associations between food biodiversity, assessed using overall, plant and animal DSR and, simultaneously, three dietary sustainability dimensions, including diet quality, environmental pressures and monetary cost.

Conducted in a large French cohort, this study showed that total DSR was associated with lower environmental pressures, as reflected, here, by lower GHGe, LO and CED, and higher overall diet quality as measured by sPNNS-GS2 and cDQI. The share of organic food in the diet was also associated with higher total DSR, while the monetary cost of the diet remained stable across DSR quintiles. Furthermore, higher total DSR was strongly positively associated with greater FG-DDS implemented in this study.

Higher plant DSR was associated with lower environmental impacts and with higher amount of plant-based food consumed. It was also positively associated with the share of organic food in the diet. In contrast, higher animal DSR was related to a higher environmental footprint, a higher amount of animal-based food in the diet and a lower share of organic food in the diet. The reduction of environmental pressures associated with total DSR seemed to be predominantly driven by the increase of plant DSR.

Mounting evidence is showing that plant-based diets have a lower environmental footprint than animal-based diets, in particular regarding GHGe^(33,34)^. Ruminants mammals are the largest emitters of GHG, followed by nonruminant mammal and poultry^(35)^.

Four studies have examined the association between total DSR and diet quality and found that a higher DSR was associated with better overall diet quality in Western countries and with better micronutrient adequacy in low- and middle-income countries^(14,15,36)^. In particular, nutritional adequacy was the highest when both DSR and food group diversity increased^(14)^. Studies assessing the relationship between higher DSR and health outcomes are scarce, but three prospective analyses have shown reduced rates of mortality as well as reduced gastrointestinal cancer risk in Europe^(13,16,36)^.

A recent study showed that total DSR, in particular animal DSR, was associated with higher GHGe and LO. Results about the environmental impacts of plant DSR were less consistent^(15)^. Many studies have focused on plant-based foods and demonstrated that diets rich in healthy plant-based foods, such as vegetables, fruits, legumes, wholegrain products and nuts, were associated with a lower risk of cancer, CVD and all-cause mortality^(37–39)^. We hypothesise that greater adherence to these healthier diets likely correlates not only with an increase in plant-based food intakes but also higher plant DSR. According to the four conceptual hypotheses cited in the introduction section, for the same amount of plant-based food consumption, increasing the plant DSR could be a relevant way to come closer to nutritional requirements, taking advantage of the synergistic effects of food products, diluting potential deleterious effects of some unhealthy foods or contaminants and stimulating gut microbial diversity through fibre intake^(13)^. This could be enhanced by higher organic food consumption, which leads to lower dietary exposure to synthetic pesticides^(40)^. These several associated factors may lead to better health outcomes^(41)^, even if the role of each of them is not easy to disentangle.

This study reveals an association between higher animal DSR and lower diet quality. However, it evaluated diet quality using sPNNS-GS2 and cDQI, which account for animal-based food consumption but overlook food biodiversity.

Overall, studies have highlighted the potential ‘co-benefits’ of plant-based diets for both human nutrition and planetary health. These diets have been associated both with reduced risks of non-communicable diseases and lower environmental pressures^(42)^. In this context, increasing plant DSR could enhance such co-benefits, reducing the risks of preventable non-communicable diseases while decreasing GHGe, LO and CED, potentially limiting global biodiversity loss and increasing ecosystem resilience, which might improve soil and plant quality, animal and human health^(43)^. It could also lead to lower exposure to contaminants such as synthetic pesticide residues, owing to the diluting concept, and the higher consumption of organic food^(44)^. Furthermore, increasing species diversity in diets could drive diversification in agricultural crops and practices. In addition, organic farming could result in higher organic matter levels and soil quality and increased species richness and evenness^(40)^. The concomitant increase of plant DSR and the share of organic food in the diet could be a relevant way to enhance the improvement of some diets’ sustainability dimensions. Nevertheless, the co-benefits of increasing overall DSR, regarding health outcomes and environmental pressures, including issues about edible and wild biodiversity preservation still warrant further attention and research.

In relation to the economic dimension, increasing plant DSR did not result in higher costs after adjustment for the amount of organic food in the diet. In the study population, we observed that plant DSR increase was concomitant to an increase in overall (animal and plant considered together) organic food consumption, consequently leading to an effective higher daily monetary cost of the overall diet. Conversely, the monetary cost of diet declined across animal DSR quintiles. This might be attributed partially to the global decline of overall organic food consumption associated with the increase of animal DSR.

The evidence so far shows that organic farming needs to be smartly combined with conventional production systems to ensure global transition towards more sustainable food systems, ensuring environment and human health and socio-economic acceptability. Even achieving these sustainability goals might appear challenging, a study suggested that a substantial fraction of the French population (nearly 20 %) managed to achieve a balance between several diet sustainability dimensions, by reducing energy density and overall energy intake and increasing plant-based foods, while remaining culturally acceptable^(45)^.

### Limitations and strengths

This study has some limitations. Participants were volunteers and thus exhibited specific characteristics as they probably paid more attention to nutrition, their health status and their dietary environmental footprint^(19)^.

Dietary data and environmental pressures’ assessment are based on FFQ data, and DSR numbering is reached after a set of allocations and conversions, which could lead to measurement errors. Indeed, the allocation factors have been assigned on the basis of 24-h dietary records, but, despite the care and rigor applied, we cannot rule out some uncertainties in the counting process. However, we applied consumption thresholds, in order not to account for marginal consumptions. The main objective of this questionnaire was to be easy to complete, while accurately capturing the relative proportion of each production method for each food item in individuals’ diets. FFQ are among the most frequently used tools when assessing food sustainability^(46)^. Although it has been shown that FFQ could result in a slight underestimation of environmental pressures, compared with other tools^(46)^, ranking of participants is considered reliable^(47)^. The Org-FFQ, used in the present work, was very detailed, composed of more than 250 items, including plant-based substitutes, and related to many species (plant or animal). This level of detail enables a more accurate estimation of the number of species consumed, while accounting for potential differences in consumption habits between organic and non-organic consumers. This FFQ was built upon an initial FFQ (without the organic scale), which has been validated (regarding relative validity and reproducibility)^(44)^, and its online version allowed an accurate estimation of the quantities consumed through many portion photographs. However, FFQ have some inherent limitations^(47)^, including items grouping and misestimation of intake. Besides, recall bias could not be entirely excluded. Moreover, in our study, organic intakes were assessed using five-point Likert scales, which probably induce measurement errors. Nevertheless, inverse associations between concentrations of some urinary pesticide biomarkers and organic food consumption, assessed using this tool, have been found in a previous work^(44)^. In a prior study, sensitivity analyses employing Monte-Carlo simulations and allocating other percentages to the ‘rarely’ modality were conducted to evaluate the influence of assigning arbitrary percentages to each frequency category on the final estimates. The variation in weights did not substantially modify individuals’ relative ranking or the analytical outcomes conclusions^(21)^. These findings do not rule out measurement errors associated with the dietary assessment method. Moreover, since the FFQ is designed to collect food consumption over the previous year, recall bias, which may vary seasonally, cannot be entirely avoided.

Finally, the acceptable level of granularity of the FFQ enables a quite reliable estimation of the share of organic food in the total diet. It should be borne in mind that, beyond providing absolute values (always prone to measurement error when based on self-reported data), the real purpose of the FFQ is to differentiate subgroups of consumers within the sample of participants, regarding species number and level of organic food consumption. In any case, while acknowledging the possibility of measurement errors, this questionnaire can be considered sufficiently valid to address our research question, particularly given that FFQ are designed to capture usual dietary intake rather than precise intake levels.

We focused on DSR as a measure of food biodiversity, since it has been demonstrated that DSR shows stronger associations and better diagnostic properties with micronutrient adequacy and health outcomes than other diversity indices, such as Shannon entropy, nutritional functional diversity or Berger–Parker index^(48)^. However, these complementary indices might provide additional insights to better understand the determinants of the sustainability of biodiverse diets in the future.

Environmental pressures have been evaluated at the production step and did not account for processing, transportation, storage and other downstream steps. However, it has been shown that the majority of environmental impacts are concentrated at the production scale^(49)^.

One of the strengths of this study is to provide results on environmental, nutrition and economic dimensions related to overall DSR, followed by distinct results when considering plant and animal biodiversity separately. Thus, we observed that a higher overall or plant DSR was associated with better nutritional and environmental metrics, whereas an inverse association was noticed for animal DSR, which does not directly align with the theoretical concepts.

Studying food sustainability remains challenging. Up to now, there is no consensus on definitions, concepts and methodological approaches to tackle the issue of food sustainability, making comparisons between studies difficult. As most of the studies, we do not consider directly the social pillar, which is the most difficult to outline and to assess^(50)^. For example, we did not evaluate the differences in cultural acceptability of more or less biodiverse diets. However, some cultural aspects are inherently considered, as this study focuses on observed dietary patterns. Likewise, despite our efforts to have an integrated approach, it remains challenging to take into account additional environmental pressures and diet quality indicators.

Finally, this study focuses on the impact of food biodiversity on environment and ecosystems without considering the links between wild biodiversity and food systems. Moreover, we do not consider varieties within species. It could be interesting to examine the potential additional co-benefits of some specific varieties within plant or animal species for human and planetary health in different settings.

This study also has many strengths. Food biodiversity is evaluated using total, plant and animal DSR separately. The results are intuitive and easy to understand for consumers, what could lead to improvements in their dietary habits. Several validated, meaningful and commonly used indicators were computed, accounting for a wide range of diet sustainability dimensions.

This study takes part about the few studies on food sustainability that integrate simultaneously environmental, nutrition/health and economic data^(50)^. To our knowledge, this is the first study to assess specifically the associations between all these metrics and food biodiversity. Moreover, the large sample size enabled us to study the associations between DSR and diet sustainability for a wide range of consumer profiles.

### Conclusion

This study examined the associations between food biodiversity and various facets of dietary sustainability. Our results indicate that plant DSR was linked to reduced environmental pressures and enhanced diet quality indicators. We may hypothesise that increasing plant DSR could provide potential co-benefits for human nutrition and the environment and should be promoted. Nonetheless, to accurately assess the potential benefits of food biodiversity for health, epidemiological studies employing causal approaches are necessary. Additionally, these findings should be validated in diverse settings beyond high-income countries, where factors such as edible species availability, agricultural practices and food affordability may significantly differ.

## Supporting information

10.1017/S1368980026102791.sm001 Berlivet et al. supplementary material Berlivet et al. supplementary material

## Data Availability

Researchers from public institutions can submit a collaboration request including information on the institution and a brief description of the project to https://collaboration@etude-nutrinet-sante.fr. All requests will be reviewed by the steering committee of the NutriNet-Santé study. A financial contribution may be requested. If the collaboration is accepted, a data access agreement will be necessary and appropriate authorisations from the competent administrative authorities may be needed. In accordance with existing regulations, no personal data will be accessible.

## Supplementary material

For supplementary material accompanying this paper visit https://doi.org/10.1017/S1368980026102791

## Table 1. Long description

The table compares characteristics of a study sample across total DSR quintiles, with five columns labeled Q1, Q2, Q3, Q4, and Q5, and various rows detailing different attributes. Row 1: Total DSR, with values < 62, 63-68, 69-72, 73-77, and > 78. Row 2: Female, with percentages 75.22, 76.23, 74.48, 73.22, and 74.60. Row 3: Male, with percentages 24.78, 23.77, 25.52, 26.78, and 25.40. Row 4: Age, years, with mean values 54.3, 54.2, 54.0, 53.6, and 51.7, and standard deviations 14.4, 14.2, 13.9, 13.5, and 13.7. Row 5: Occupational status, with various percentages for Unemployed (others), Unemployed, Student, Retired, Farmer, self-employed, Intellectual profession, Employee, Manual worker, and Intermediate profession. Row 6: Educational level, with percentages for < High school, High school, and > High school. Row 7: Monthly income per household unit (€), with percentages for < 1200, 1200-2300, 2300-3700, > 3700, and Unwilling to answer. Row 8: Smoking status, with percentages for Non-smoker, Former smoker, and Current smoker. Row 9: Physical activity level (MET-min/week), with percentages for Missing data, High (> 1500), Moderate (600-1500), and Low (< 600). Row 10: Energy intake without alcohol, with mean values 1800, 1901, 1965, 1994, and 2048, and standard deviations 619, 598, 602, 601, and 619.

Navigate back to Table 1..
