# Supplementary material for: Sustainability dimensions of diets with varying levels of food biodiversity in the NutriNet-Santé study
Source: Public Health Nutr. 2026 May 28;29(1):e135. doi: 10.1017/S1368980026102791 (PMC13434557; doi:10.1017/S1368980026102791)
Supplement: Supplementary file 1 [file S1368980026102791sup001.docx]

**Supplemental Files**

Supplemental Method: Computation of environmental data.............................................................................2

Supplemental Table 0.1: Cumulative energy demand posts and calculation method by production

Supplemental Table 0.2: Points of GHG emissions and calculation method by production

Supplemental Table 0.3: Examples of aggregation of some specific agricultural products

Table S1: Food consumption (g/day) across total DSR quintiles (cut-off 5%)^1^, adjusted for age, sex, and alcohol-free energy intake..................................................................................................................................8

Table S2: Food consumption (g/day) across plant DSR quintiles (cut-off 5%)^1^, adjusted for age, sex, alcohol-free energy intake and animal DSR.......................................................................................................9

Table S3: Food consumption (g/day) across animal DSR quintiles (cut-off 5%)^1^, adjusted for age, sex, alcohol-free energy intake and plant DSR.......................................................................................................10

Table S4: Sustainability indicators and total DSR (cut-off 5%)^1^ assessed by quintiles or as a continuous variable.............................................................................................................................................................11

Table S5: Sustainability indicators and total DSR (cut-off 5%)^1^ assessed by quintiles or as a continuous variable, model adjusted for age, sex, alcohol-free energy intake and the amount of plant protein in the diet....................................................................................................................................................................13

Table S6: Sustainability indicators and plant DSR (cut-off 5%)^1^ assessed by quintiles or as a continuous variable.............................................................................................................................................................14

Table S7: Sustainability indicators and animal DSR (cut-off 5%)^1^ assessed by quintiles or as a continuous variable.............................................................................................................................................................16

Table S8: Characteristics of the study sample by total DSR quintiles (cut-off 5g)^2^........................................18

Table S9: Sustainability indicators and total DSR (cut-off 5g)^2^ assessed by quintiles or as a continuous variable.............................................................................................................................................................19

Table S10: Sustainability indicators and total DSR (cut-off 5g)^2^ assessed by quintiles or as a continuous variable, model adjusted for age, sex, alcohol-free energy intake and plant protein in the diet......................21

Table S11: Sustainability indicators and plant DSR (cut-off 5g)^2^ assessed by quintiles or as a continuous variable.............................................................................................................................................................22

Table S12: Sustainability indicators and animal DSR (cut-off 5g)^2^ assessed by quintiles or as a continuous variable............................................................................................................................................................24

Figure S1: Sustainability indicators and plant DSR (cut-off 5%)^1^ assessed by quintiles or as a continuous variable, further adjusted for the share of organic food in the diet.................................................................25

Figure S2: Sustainability indicators and animal DSR (cut-off 5%)^1^ assessed by quintiles or as a continuous variable, further adjusted for the share of organic food in the diet.................................................................26

^1^ species accounting for less than 5% (in grams) of any of the 33 food groups to which they pertain, are not accounted for in the DSR computation.

^2^ species accounting for less than 5g are not accounted for in the DSR computation.

**Supplemental Method : Computation of environmental data**

Evaluation of the agricultural production stage

Data sources and perimeter of analysis

Environmental data were derived from the French diagnostic tool DIALECTE ^(1)^. DIALECTE is a comprehensive tool developed by Solagro which aims to describe farming systems and to assess the environmental performance of farms using a global approach at the farm scale. To date, the DIALECTE database includes information from 2,086 farms with various agricultural production systems, in particular organic farming, throughout France. A total of 46% of farms included in the DIALECTE database followed certified organic practices.

Information collected by DIALECTE allowed an estimation of the environmental impacts at the farm level of 60 raw agricultural products using the life cycle assessment (LCA) method. Here, the perimeter of LCA was limited to the agricultural production step. Of note, the upstream processes were included in the assessment such as the production of inputs or energy provision. Conditioning, transport, processing, storage and recycling were excluded from the analysis. This limitation should be considered as relative since most environmental impacts generally stem from the production step ^(2,3)^. This is less true for alcoholic beverages as regards energy consumption and CO2 emissions since they require a high level of processing and bottling, or for fruit and vegetables transported by plane.

DIALECTE was chosen due to its great diversity of data based on real French farms from various types of farming systems. Apart from the Agribalyse project ^(4)^ in which organic data are scarce, to our knowledge, DIALECTE is to date the only French database that covers such a large panel data for both organic and conventional agricultural products, making it possible the calculation of LCA-based indicators for the whole diet.

Assessment of environmental indicators at farm scale

- Cumulative energy demand (CED)

Cumulative energy demand (in MJ) included consumption of renewable and non-renewable energy as defined by the CED method ^(5)^, commonly used in LCA. The calculation system used was based on the Dia’terre^®^ instrument ^(6)^. The use of renewable energies is limited at the farm level, as a very low percentage of farms in DIALECTE have solar dryers, water panels or biogas plant which reduce the consumption of non-renewable energies.

**Supplemental Table 0.1.** shows the different cumulative energy demand items and the nature of the data used. Most of the energy demand positions were evaluated at the farm level. Allocations (with generic energy demand coefficient) were computed to share the energetic consumption between vegetable production and livestock building.

**Supplemental Table 0.1.: Cumulative energy demand posts and calculation method by production**

| Cumulative energy demand posts | Survey data | Attribution |
| --- | --- | --- |
| *Directs energy (without irrigation)* | |  |
| Fuel^1^ (Fuel, lubricants, diesel and petrol) | Data at the farm level | Allocation of energy consumption in proportion to the theoretical consumption of crops and livestock |
| Gas (propane, butane, natural gas) |  |  |
| Electricity |  |  |
| *Direct energy for irrigation* | |  |
| Fuel^1^ | Data at the farm level | Prorated calculation of attribution in function of theoretic consumption between vegetable production and livestock building |
| Electricity^1^ |  |  |
| *Indirect energy* | | |
| Livestock and veterinary products | Data at the farm level | Non-included |
| Mechanization |  | Prorated calculation of attribution for vegetable production, in function of land occupation |
| Building (<30 years) |  |  |
| Plastic sheeting |  |  |
| Preservatives or silage additives |  |  |
| Feed |  | Prorated calculation of attribution for animals in function of the consumption of forage and concentrates |
| Fertilizer | Data at the crop level | N/A |
| Phytosanitary products |  |  |

Abbreviation: N/A, Not applicable. ^1^Including consumption for third parties

- Greenhouse gas emissions (GHGEs)

The GHGE referred to the definition established by the Intergovernmental Panel on Climate Change, *i.e.* the quantity of three GHGs (carbon dioxide CO_2_, methane CH_4_ and nitrous oxide N_2_O), weighted by their 100-year Global Warming Potential, in kg eqCO_2_. Their calculation was based on the methodology developed in the tool Dia’terre^®^ ^(7)^.

**Supplemental Table 0.2** shows the different posts of GHGEs taken into account in the LCA. Allocations with generic energy demand coefficients were used to share the emissions between vegetable production and livestock building.

**Supplemental Table 0.2: Points of GHG emissions and calculation method by production**

| Source of GHGEs | Survey data | Allocation |
| --- | --- | --- |
| *Direct energy* | | |
| Direct energy without irrigation  (fuel, gas, electricity) | Data at farm level | Prorated calculation in function of theoretic consumption between vegetable production and livestock building |
| Direct energy for irrigation  (fuel, electricity) | Data at farm level | Prorated calculation in function of water consumption by crop |
| *Indirect energy* | | |
| Mechanization, plastics, building (< 30 years), preservatives silage and veterinary products | Data at farm level | Prorated calculation for vegetable production, in function of land occupation. |
| Fertilizers and pesticides | Data at crop level | N/A |
| Feed | Data at farm level | Prorated calculation for animals in function of the consumption of forage and concentrates. |
| **Diffuse emissions** | | |
| Enteric fermentation | Data by domestic animal category | N/A |
| Direct emission of N_2_O (Application of nitrogen fertilizers and manure, nitrogen excreted during grazing, mineralization of crop residues. | Data at crop level |  |
| Indirect emission of N_2_O (atmospheric deposition, runoff and leaching of reactive nitrogen) |  |  |
| **Carbone storage in soil and in trees** |  | Non-included |

Abbreviation: N/A, Not applicable

- Land occupation

Land occupation corresponded to the area required to produce raw agricultural products, without taking into account the duration of the land use. It is expressed as the inverse of yield, in m^2^/kg. This indicator differs from the LCA indicator *land use,* expressed in m^2^.kg^-1^.year, which reflects the surface of land occupied over a fixed period of time. Both indicators are similar when the production period is one year, which corresponds to most agricultural production cycles.

For 29 types of vegetables, the impacts were calculated using the average yield between 2010 and 2015 from the agricultural annual French statistics, rather than survey data. Indeed, most vegetables are produced on small areas and yield calculation over one hectare may lead to discrepancies. As the French statistics provided data for conventional farming only, we used a rebate coefficient for organic farming of 23% for all vegetable crops, excepted for strawberries (41%) and salads (14%) ^(8)^. Also, for vegetable crops, impacts per hectare were computed depending on the mean of production: heated or cold greenhouses or field grown ^(8)^.

For animal production, the indicator was calculated as the sum of the land areas needed for pasture and feed production, including purchased feed while neglecting areas related to livestock farm facilities.

Aggregation for particular products

Some agricultural products available in DIALECTE but not in the BioNutriNet database were aggregated into one single product reference (**Supplemental** **Table 0.3.**) by using a ponderation based on the Annual Agricultural Survey from the French Ministry of Agriculture ^(9)^.

**Supplemental Table 0.3.: Examples of aggregation of some specific agricultural products**

| Aggregated agricultural product | DIALECTE agricultural product | Proportion |
| --- | --- | --- |
| Olive | Black olive | 0.5 |
|  | Green olive | 0.5 |
| Chicken | Standard chicken | 0.88 |
|  | Labelled chicken | 0.12 |
| Bovine meat | From cattle rearing and fattening farms | 0.66 |
|  | From dairy cattle | 0.34 |
| Sheep meat | Sheep fattening | 0.9 |
|  | Dairy Sheep | 0.1 |

Moreover, tomatoes produced under heated greenhouses were considered as ‘cherry tomatoes’ or ‘fresh tomatoes’ and tomatoes produced in open fields as ‘cooked tomatoes’ and ‘tomato pulp’.

Determination of the reference value

Using the methodology described above, we obtained environmental impacts for 60 raw agricultural products from 2,086 farms surveyed with DIALECTE, of which 46% were conducted according to organic principles. Regarding organic farming, the number of available data ranged from 2 (duck) to 355 (beef meat) (interquartile range (IQR): 34; 348). Concerning conventional farming, the number of available data ranged from 1 (nuts) to 408 (soft wheat) (IQR: 40; 348). Medians were taken as central values since outliers are relatively common in this type of survey.

For products with a high contribution to the diet and for which data were not available in DIALECTE, such as seafood (N=6), honey, tropical products (N=12) and for some products with few references in DIALECTE (N=13), impacts were assessed using published literature data, and Agribalyse® data in particular ^(4)^.

Finally, the three environmental indicators were calculated for 92 raw agricultural products, conventional and organic, 60 from DIALECTE and 32 from literature searches.

Conversion from raw agricultural products to food items

As our goal was to assess the environmental impacts of diets, we conducted a set of conversions to obtain data about food items.

Conversion from raw agricultural products to ingredients

As a first step, to obtain values for each indicator and ingredient from agricultural raw products, economic attributions by co-products were applied followed by the allocations of mass, cooking, and edibility coefficients in order to obtain indicator values for the ingredient as consumed, using data from literature and NutriNet-santé composition database (for the allocations of cooking and edibility coefficients). The same conversion factors were used for organic and conventional products.

Overall, a total of 766 ingredients are components of the 264 food items of the FFQ (Food Frequency Questionnaire). For feasibility reasons, the environmental impacts of the ingredients which accounted for at least 5% of the recipe were computed, corresponding to 442 ingredients.

Data were unavailable for 59 ingredients which accounted for more than 5% in at least one recipe (*e.g.* tropical areas, certain alcoholic beverages, grains or yeast). Their environmental impacts were considered as null.

Conversion from ingredients to food items

Each of the 264 items of the FFQ was composed of sub-items (or foods). For example, the item pasta was composed of many sub-items (vermicelli, pasta, Chinese noodles, egg pasta, fresh pasta, and egg and fresh pasta spinach flavor). The composition of each item was the result of the multiplication of gender-specific consumption frequency of each sub-item (determined using the 24-h dietary records of NutriNet-Santé) by the proportion of each ingredient constituting the sub-item.

About 75% of food items had more than 98.8% of their composition covered by the 442 ingredients. When an ingredient was missing for a given item, environmental impacts were ‘standardized’ for 100g. The ingredients concerned by the standardization were fish products, jam, cheese and dressings.

Finally, we obtained environmental impacts for most of the 264 food items in organic and conventional forms. No data were available for 22 items for CED, 21 items for GHGE and 25 items for land occupation. Missing data included the following food items: water, certain alcoholic beverages, tropical fruit or vegetables, grains and certain oils, considered as marginal.

Table S1: Food consumption (g/day) across total DSR quintiles (cut-off 5%)^1^ adjusted for age, sex, and alcohol-free energy intake

|  | Q1 | Q2 | Q3 | Q4 | Q5 | *P*-trend^2^ |
| --- | --- | --- | --- | --- | --- | --- |
| Total DSR | <62 | 63-68 | 69-72 | 73-77 | >78 |  |
| Bread | 55.66 (0.70) | 55.81 (0.70) | 53.80 (0.66) | 47.60 (0.71) | 37.93 (0.71) | <0.0001 |
| Butter | 6.65 (0.09) | 6.70 (0.09) | 6.82 (0.08) | 6.60 (0.09) | 6.01 (0.09) | <0.0001 |
| Cereals | 80.20 (0.84) | 80.94 (0.84) | 82.25 (0.79) | 80.95 (0.85) | 81.34 (0.85) | 0.4 |
| Cheese | 41.97 (0.54) | 42.91 (0.54) | 42.89 (0.51) | 40.09 (0.54) | 38.39 (0.55) | <0.0001 |
| Cookies | 9.80 (0.20) | 10.13 (0.20) | 9.62 (0.19) | 9.39 (0.20) | 8.84 (0.20) | <0.0001 |
| Dairy products | 178.59 (1.97) | 162.69 (1.96) | 154.15 (1.85) | 152.22 (1.98) | 142.50 (1.99) | <0.0001 |
| Dressings | 7.00 (0.10) | 7.40 (0.10) | 7.27 (0.10) | 7.32 (0.11) | 6.87 (0.11) | 0.31 |
| Fast food | 29.02 (0.44) | 32.48 (0.44) | 34.64 (0.41) | 34.05 (0.44) | 35.38 (0.44) | <0.0001 |
| Milky desserts | 10.28 (0.33) | 12.31 (0.33) | 12.69 (0.31) | 12.62 (0.33) | 11.66 (0.33) | 0.004 |
| Non-alcoholic drinks | 780.99 (6.42) | 774.63 (6.39) | 801.15 (6.05) | 813.75 (6.47) | 849.52 (6.50) | <0.0001 |
| Oils | 18.92 (0.19) | 19.77 (0.19) | 19.54 (0.18) | 19.94 (0.19) | 21.14 (0.19) | <0.0001 |
| Potatoes | 22.89 (0.27) | 22.44 (0.27) | 22.11 (0.26) | 21.40 (0.27) | 20.01 (0.28) | <0.0001 |
| Poultry | 23.75 (0.30) | 22.79 (0.30) | 22.52 (0.29) | 21.96 (0.31) | 20.31 (0.31) | <0.0001 |
| Snacks | 8.45 (0.18) | 9.57 (0.18) | 9.74 (0.17) | 10.09 (0.18) | 10.93 (0.18) | <0.0001 |
| Vegetables | 283.43 (2.42) | 264.58 (2.41) | 261.88 (2.28) | 270.13 (2.44) | 288.48 (2.45) | 0.04 |
| Wholegrain products | 48.32 (0.90) | 49.90 (0.90) | 52.70 (0.85) | 60.36 (0.91) | 71.80 (0.91) | <0.0001 |
| Eggs | 11.80 (0.15) | 11.20 (0.15) | 11.18 (0.14) | 11.12 (0.15) | 11.65 (0.15) | 0.44 |
| Fat | 3.34 (0.06) | 3.51 (0.06) | 3.49 (0.06) | 3.55 (0.06) | 3.44 (0.06) | 0.24 |
| Alcohol | 97.26 (1.85) | 103.85 (1.84) | 107.51 (1.74) | 100.60 (1.86) | 95.46 (1.87) | 0.25 |
| Fish | 44.60 (0.55) | 44.85 (0.55) | 45.82 (0.52) | 47.75 (0.55) | 48.26 (0.56) | <0.0001 |
| Fruit juice | 84.72 (1.48) | 84.90 (1.48) | 82.56 (1.40) | 83.59 (1.49) | 79.85 (1.50) | 0.02 |
| Grains | 2.65 (0.12) | 2.53 (0.12) | 2.80 (0.11) | 3.70 (0.12) | 5.75 (0.12) | <0.0001 |
| Fruit | 338.77 (3.11) | 284.67 (3.09) | 270.79 (2.93) | 267.64 (3.13) | 271.12 (3.15) | <0.0001 |
| Milk | 60.65 (1.75) | 53.98 (1.74) | 61.24 (1.65) | 58.87 (1.76) | 51.06 (1.77) | 0.01 |
| Milk soy | 22.99 (1.17) | 18.96 (1.17) | 18.13 (1.11) | 27.86 (1.18) | 45.49 (1.19) | <0.0001 |
| Red meat | 46.80 (0.53) | 46.51 (0.53) | 45.12 (0.50) | 42.64 (0.53) | 37.30 (0.54) | <0.0001 |
| Sodas | 57.26 (1.38) | 45.57 (1.38) | 40.85 (1.30) | 36.24 (1.39) | 29.49 (1.40) | <0.0001 |
| Soy-based products | 5.04 (0.25) | 3.81 (0.25) | 3.74 (0.23) | 4.30 (0.25) | 7.09 (0.25) | <0.0001 |
| Soup | 83.47 (1.29) | 81.73 (1.28) | 85.99 (1.22) | 88.28 (1.30) | 94.55 (1.31) | <0.0001 |
| Sweets | 44.59 (0.46) | 48.05 (0.45) | 48.88 (0.43) | 50.47 (0.46) | 50.72 (0.46) | <0.0001 |
| White meat | 73.40 (0.62) | 72.62 (0.61) | 71.53 (0.58) | 68.40 (0.62) | 59.99 (0.62) | <0.0001 |
| Legumes | 13.82 (0.39) | 15.54 (0.39) | 15.50 (0.37) | 16.80 (0.39) | 19.99 (0.40) | <0.0001 |
| Dried fruits | 2.74 (0.10) | 3.24 (0.10) | 3.45 (0.10) | 3.99 (0.10) | 4.94 (0.10) | <0.0001 |

Values are mean (standard deviation). Q: quintile, DSR: dietary species richness

^1^ species accounting for less than 5% (in grams) of any of the 33 food groups to which they pertain, are not accounted for in the DSR computation.

^2^ P-trend for contrasts across quintiles.

Table S2: Food consumption (g/day) across plant DSR quintiles (cut-off 5%)^1^ adjusted for age, sex, alcohol-free energy intake and animal DSR

|  | Q1 | Q2 | Q3 | Q4 | Q5 | P-trend^2^ |
| --- | --- | --- | --- | --- | --- | --- |
| Plant DSR | <42 | 41-45 | 46-49 | 50-54 | >55 |  |
| Bread | 61.81 (0.69) | 56.13 (0.69) | 52.37 (0.69) | 44.91 (0.73) | 35.61 (0.68) | <0.0001 |
| Butter | 7.31 (0.09) | 6.86 (0.09) | 6.59 (0.09) | 6.36 (0.09) | 5.67 (0.09) | <0.0001 |
| Cereals | 79.65 (0.83) | 79.20 (0.83) | 82.14 (0.83) | 82.24 (0.88) | 82.74 (0.82) | <0.0001 |
| Cheese | 43.48 (0.53) | 43.97 (0.53) | 42.45 (0.53) | 39.68 (0.57) | 36.77 (0.53) | <0.0001 |
| Cookies | 11.09 (0.19) | 9.85 (0.19) | 9.81 (0.19) | 8.99 (0.21) | 7.97 (0.19) | <0.0001 |
| Dairy products | 182.09 (1.93) | 161.13 (1.93) | 157.98 (1.93) | 152.73 (2.06) | 135.24 (1.92) | <0.0001 |
| Dressings | 7.20 (0.10) | 7.49 (0.10) | 7.26 (0.10) | 7.20 (0.11) | 6.75 (0.10) | <0.0001 |
| Fast food | 33.65 (0.43) | 33.15 (0.43) | 33.36 (0.43) | 33.03 (0.46) | 32.45 (0.42) | 0.06 |
| Milky desserts | 13.69 (0.32) | 12.58 (0.32) | 11.84 (0.32) | 11.38 (0.34) | 10.06 (0.32) | <0.0001 |
| Non-alcoholic drinks | 755.55 (6.31) | 778.53 (6.31) | 810.78 (6.31) | 826.82 (6.73) | 850.23 (6.27) | 0.0002 |
| Oils | 17.62 (0.18) | 19.50 (0.18) | 19.58 (0.18) | 20.45 (0.20) | 22.18 (0.18) | 0.0003 |
| Potatoes | 23.20 (0.27) | 22.10 (0.27) | 21.83 (0.27) | 21.54 (0.29) | 20.23 (0.27) | 0.0004 |
| Poultry | 25.04 (0.30) | 23.22 (0.30) | 22.64 (0.30) | 21.31 (0.32) | 19.07 (0.30) | 0.001 |
| Snacks | 6.92 (0.17) | 9.24 (0.17) | 9.86 (0.17) | 10.71 (0.18) | 12.17 (0.17) | 0.001 |
| Vegetables | 245.24 (2.32) | 257.13 (2.31) | 271.19 (2.31) | 283.19 (2.47) | 311.55 (2.30) | 0.001 |
| Wholegrain products | 40.37 (0.88) | 49.19 (0.88) | 54.64 (0.88) | 63.49 (0.93) | 75.54 (0.87) | 0.001 |
| Eggs | 11.46 (0.15) | 11.22 (0.15) | 11.32 (0.15) | 11.10 (0.16) | 11.76 (0.15) | 0.31 |
| Fat | 3.49 (0.06) | 3.48 (0.06) | 3.38 (0.06) | 3.49 (0.06) | 3.50 (0.06) | 0.89 |
| Alcohol | 104.36 (1.82) | 107.57 (1.81) | 104.57 (1.81) | 96.97 (1.94) | 91.67 (1.80) | <0.0001 |
| Fish | 44.70 (0.54) | 44.76 (0.54) | 47.34 (0.54) | 47.66 (0.58) | 46.89 (0.54) | <0.0001 |
| Fruit juice | 86.04 (1.46) | 85.28 (1.46) | 80.64 (1.46) | 84.96 (1.56) | 78.89 (1.45) | 0.002 |
| Grains | 1.11 (0.12) | 2.15 (0.12) | 3.02 (0.12) | 4.20 (0.12) | 6.91 (0.12) | <0.0001 |
| Fruit | 290.68 (3.02) | 281.29 (3.01) | 281.75 (3.01) | 287.14 (3.22) | 290.99 (3.00) | 0.5 |
| Milk | 77.90 (1.70) | 59.99 (1.70) | 56.73 (1.70) | 52.35 (1.82) | 38.45 (1.69) | <0.0001 |
| Milk soy | 2.45 (1.10) | 13.90 (1.10) | 20.98 (1.10) | 31.30 (1.17) | 64.07 (1.09) | <0.0001 |
| Red meat | 52.09 (0.51) | 47.90 (0.51) | 43.87 (0.51) | 40.81 (0.55) | 33.60 (0.51) | <0.0001 |
| Sodas | 59.53 (1.36) | 44.21 (1.36) | 40.60 (1.36) | 37.34 (1.45) | 27.17 (1.35) | <0.0001 |
| Soy-based products | -0.16 (0.23) | 2.75 (0.23) | 4.13 (0.23) | 5.98 (0.24) | 11.30 (0.23) | <0.0001 |
| Soup | 80.96 (1.27) | 81.32 (1.27) | 87.61 (1.27) | 89.10 (1.35) | 95.02 (1.26) | <0.0001 |
| Sweets | 48.21 (0.45) | 48.22 (0.45) | 48.45 (0.45) | 49.08 (0.48) | 48.75 (0.44) | 0.18 |
| White meat | 81.09 (0.59) | 74.30 (0.59) | 70.57 (0.59) | 65.44 (0.63) | 54.45 (0.59) | <0.0001 |
| Legumes | 8.28 (0.37) | 13.92 (0.37) | 15.89 (0.37) | 19.33 (0.40) | 24.51 (0.37) | <0.0001 |
| Dried fruits | 1.66 (0.10) | 2.99 (0.10) | 3.76 (0.10) | 4.43 (0.10) | 5.58 (0.10) | <0.0001 |

Values are mean (standard deviation). Q: quintile, DSR: dietary species richness

^1^ species accounting for less than 5% (in grams) of any of the 33 food groups to which they pertain, are not accounted for in the DSR computation.

^2^ P-trend for contrasts across quintiles.

Table S3: Food consumption (g/day) across animal DSR quintiles (cut-off 5%)^1^ adjusted for age, sex, alcohol-free energy intake and plant DSR

|  | Q1 | Q2 | Q3 | Q4 | Q5 | P-trend^2^ |
| --- | --- | --- | --- | --- | --- | --- |
| Animal DSR | <19 | 20-21 | 22-23 | 24-25 | >26 |  |
| Bread | 39.71 (0.70) | 51.67 (0.74) | 54.85 (0.61) | 53.52 (0.70) | 51.05 (0.74) | <0.0001 |
| Butter | 5.41 (0.09) | 6.42 (0.09) | 6.97 (0.08) | 6.98 (0.09) | 6.99 (0.09) | <0.0001 |
| Cereals | 83.34 (0.84) | 79.44 (0.89) | 79.76 (0.74) | 81.63 (0.85) | 81.86 (0.89) | 0.78 |
| Cheese | 38.15 (0.54) | 43.63 (0.57) | 42.79 (0.48) | 42.03 (0.54) | 39.70 (0.57) | 0.41 |
| Cookies | 7.46 (0.20) | 9.38 (0.21) | 9.96 (0.17) | 10.28 (0.20) | 10.76 (0.21) | <0.0001 |
| Dairy products | 153.15 (1.96) | 168.34 (2.08) | 163.38 (1.73) | 157.30 (1.97) | 146.68 (2.08) | <0.0001 |
| Dressings | 6.62 (0.10) | 6.98 (0.11) | 7.45 (0.09) | 7.49 (0.11) | 7.29 (0.11) | <0.0001 |
| Fast food | 25.41 (0.43) | 32.22 (0.46) | 34.37 (0.38) | 36.03 (0.44) | 37.85 (0.46) | <0.0001 |
| Milky desserts | 5.83 (0.33) | 11.33 (0.35) | 13.31 (0.29) | 14.21 (0.33) | 14.98 (0.35) | <0.0001 |
| Non-alcoholic drinks | 832.55 (6.42) | 803.59 (6.81) | 792.52 (5.66) | 797.06 (6.45) | 794.08 (6.82) | <0.0001 |
| Oils | 22.54 (0.19) | 19.93 (0.20) | 18.97 (0.17) | 19.03 (0.19) | 18.83 (0.20) | <0.0001 |
| Potatoes | 21.28 (0.27) | 21.78 (0.29) | 22.45 (0.24) | 21.99 (0.27) | 21.21 (0.29) | 0.93 |
| Poultry | 19.95 (0.30) | 23.68 (0.32) | 23.36 (0.27) | 22.44 (0.30) | 21.86 (0.32) | 0.001 |
| Snacks | 12.55 (0.17) | 9.81 (0.19) | 9.13 (0.15) | 8.55 (0.18) | 8.71 (0.19) | <0.0001 |
| Vegetables | 338.01 (2.38) | 271.48 (2.52) | 255.94 (2.10) | 252.57 (2.39) | 249.52 (2.53) | <0.0001 |
| Wholegrain products | 69.94 (0.89) | 56.33 (0.94) | 51.26 (0.79) | 51.00 (0.89) | 54.57 (0.95) | <0.0001 |
| Eggs | 12.29 (0.15) | 11.28 (0.16) | 11.42 (0.13) | 11.01 (0.15) | 10.81 (0.16) | <0.0001 |
| Fat | 3.28 (0.06) | 3.31 (0.06) | 3.59 (0.05) | 3.56 (0.06) | 3.54 (0.06) | 0.0001 |
| Alcohol | 90.40 (1.85) | 97.75 (1.96) | 104.26 (1.63) | 106.34 (1.86) | 106.35 (1.96) | <0.0001 |
| Fish | 46.75 (0.55) | 45.96 (0.58) | 46.89 (0.48) | 45.89 (0.55) | 45.32 (0.58) | 0.11 |
| Fruit juice | 80.65 (1.48) | 79.21 (1.57) | 84.67 (1.31) | 84.87 (1.49) | 85.65 (1.58) | 0.001 |
| Grains | 6.63 (0.12) | 3.42 (0.13) | 2.74 (0.10) | 2.27 (0.12) | 2.23 (0.13) | <0.0001 |
| Fruit | 360.50 (3.08) | 296.35 (3.27) | 271.27 (2.72) | 258.54 (3.10) | 244.61 (3.28) | <0.0001 |
| Milk | 30.86 (1.73) | 44.62 (1.83) | 57.28 (1.52) | 69.31 (1.74) | 86.51 (1.84) | <0.0001 |
| Milk soy | 63.41 (1.13) | 24.23 (1.20) | 17.48 (1.00) | 13.32 (1.14) | 13.58 (1.20) | <0.0001 |
| Red meat | 34.13 (0.52) | 46.22 (0.55) | 46.29 (0.46) | 46.62 (0.53) | 45.45 (0.56) | <0.0001 |
| Sodas | 39.79 (1.38) | 45.13 (1.46) | 43.61 (1.22) | 43.16 (1.39) | 37.55 (1.47) | 0.16 |
| Soy-based products | 14.30 (0.24) | 3.80 (0.25) | 2.34 (0.21) | 1.62 (0.24) | 1.79 (0.25) | <0.0001 |
| Soup | 90.33 (1.29) | 87.13 (1.37) | 85.56 (1.14) | 83.11 (1.30) | 87.98 (1.37) | 0.04 |
| Sweets | 41.56 (0.45) | 47.66 (0.48) | 50.05 (0.40) | 51.58 (0.46) | 51.75 (0.48) | <0.0001 |
| White meat | 54.87 (0.60) | 72.03 (0.64) | 74.42 (0.53) | 73.03 (0.61) | 71.55 (0.64) | <0.0001 |
| Legumes | 25.96 (0.38) | 14.96 (0.40) | 13.59 (0.34) | 13.38 (0.38) | 13.65 (0.41) | <0.0001 |
| Dried fruits | 5.62 (0.10) | 3.76 (0.11) | 3.05 (0.09) | 3.11 (0.10) | 2.81 (0.11) | <0.0001 |

Values are mean (standard deviation). Q: quintile, DSR: dietary species richness

^1^: species accounting for less than 5% (in grams) of any of the 33 food groups to which they pertain, are not accounted for in the DSR computation.

^2^: P-trend for contrasts across quintiles.

Table S4: Sustainability indicators and total DSR (cut-off 5%)^1^ assessed by quintiles or as a continuous variable

|  |  | Q1 (n=5758; 2-62) | Q2 (n=5759; 63-68) | Q3 (n=6418; 69-72) | Q4 (n=5620; 73-77) | Q5 (n=5618; 78-103) | *P*-trend^4^ | Per 10 species increment | *P*-value^5^ |
| --- | --- | --- | --- | --- | --- | --- | --- | --- | --- |
| **GHG emissions (kg eqCO_2_ / day)** | | | |  |  |  |  |  |  |
|  | Model 1^2^ | 4.22 (4.17; 4.27) | 4.22 (4.16; 4.27) | 4.16 (4.11; 4.21) | 4.00 (3.95; 4.06) | 3.65 (3.60; 3.70) | <0.0001 |  |  |
|  | Model 2^3^ | 4.21 (4.15; 4.26) | 4.21 (4.16; 4.27) | 4.15 (4.10; 4.20) | 4.00 (3.95; 4.05) | 3.67 (3.62; 3.73) | <0.0001 | -0.17 (-0.19; -0.14) | <0.0001 |
| **Land occupation (m^2^ / day)** | | |  |  |  |  |  |  |  |
|  | Model 1^2^ | 10.83 (10.69; 10.98) | 10.93 (10.79; 11.08) | 10.81 (10.67; 10.94) | 10.55 (10.40; 10.70) | 9.87 (9.72; 10.02) | <0.0001 |  |  |
|  | Model 2^3^ | 10.78 (10.64; 10.93) | 10.92 (10.78; 11.07) | 10.80 (10.66; 10.93) | 10.54 (10.40; 10.69) | 9.95 (9.80; 10.10) | <0.0001 | -0.27 (-0.33; -0.20) | <0.0001 |
| **Energy demand (MJ / day)** | | |  |  |  |  |  |  |  |
|  | Model 1^2^ | 18.51 (18.38; 18.65) | 18.06 (17.92; 18.20) | 17.83 (17.70; 17.96) | 17.37 (17.24; 17.51) | 16.31 (16.17; 16.45) | <0.0001 |  |  |
|  | Model 2^3^ | 18.46 (18.32; 18.59) | 18.03 (17.90; 18.17) | 17.81 (17.69; 17.94) | 17.37 (17.23; 17.51) | 16.42 (16.29; 16.56) | <0.0001 | -0.68 (-0.74; -0.62) | <0.0001 |
| **pRecipe** | |  |  |  |  |  |  |  |  |
|  | Model 1^2^ | 0.72 (0.71; 0.72) | 0.72 (0.71; 0.73) | 0.71 (0.70; 0.72) | 0.69 (0.68; 0.70) | 0.64 (0.63; 0.65) | <0.0001 |  |  |
|  | Model 2^3^ | 0.71 (0.70; 0.72) | 0.72 (0.71; 0.73) | 0.71 (0.70; 0.72) | 0.69 (0.68; 0.70) | 0.65 (0.64; 0.66) | <0.0001 | -0.02 (-0.03; -0.02) | <0.0001 |
| **comprehensive Dietary Quality Index (cDQI)** | | | |  |  |  |  |  |  |
|  | Model 1^2^ | 49.86 (49.63; 50.10) | 50.59 (50.35; 50.82) | 51.16 (50.94; 51.38) | 52.06 (51.83; 52.30) | 53.91 (53.67; 54.14) | <0.0001 |  |  |
|  | Model 2^3^ | 49.76 (49.54; 49.98) | 50.40 (50.18; 50.62) | 51.09 (50.88; 51.30) | 52.12 (51.90; 52.34) | 54.23 (54.00; 54.45) | <0.0001 | 1.43 (1.33; 1.53) | <0.0001 |
| **simplified PNNS Guidelines Score – 2^nd^ version (sPNNS-GS2)** | | | | |  |  |  |  |  |
|  | Model 1^2^ | 2.41 (2.33; 2.49) | 2.45 (2.37; 2.53) | 2.51 (2.43; 2.58) | 2.84 (2.77; 2.92) | 3.46 (3.38; 3.54) | <0.0001 |  |  |
|  | Model 2^3^ | 2.41 (2.33; 2.48) | 2.42 (2.35; 2.50) | 2.50 (2.43; 2.57) | 2.85 (2.78; 2.93) | 3.49 (3.41; 3.56) | <0.0001 | 0.33 (0.30; 0.37) | <0.0001 |
| **Share of organic food in the diet (g/day, %)** | | | |  |  |  |  |  |  |
|  | Model 1^2^ | 0.23 (0.22; 0.24) | 0.25 (0.24; 0.26) | 0.27 (0.26; 0.28) | 0.32 (0.31; 0.33) | 0.42 (0.41; 0.42) | <0.0001 |  |  |
|  | Model 2^3^ | 0.23 (0.22; 0.24) | 0.25 (0.24; 0.26) | 0.27 (0.26; 0.27) | 0.32 (0.31; 0.33) | 0.42 (0.41; 0.43) | <0.0001 | 0.06 (0.06; 0.06) | <0.0001 |
| **Food Groups-based-Dietary Diversity Score (FG-DDS)** | | | | |  |  |  |  |  |
|  | Model 1^2^ | 8.46 (8.41; 8.51) | 8.79 (8.74; 8.84) | 8.97 (8.92; 9.02) | 9.18 (9.13; 9.23) | 9.46 (9.41; 9.51) | <0.0001 |  |  |
|  | Model 2^3^ | 8.44 (8.39; 8.48) | 8.76 (8.71; 8.81) | 8.96 (8.91; 9.00) | 9.19 (9.14; 9.24) | 9.52 (9.47; 9.57) | <0.0001 | 0.38 (0.36; 0.40) | <0.0001 |
| **Monetary cost of the diet (€ / day)** | | | |  |  |  |  |  |  |
|  | Model 1^2^ | 7.84 (7.79; 7.90) | 7.63 (7.58; 7.69) | 7.60 (7.54; 7.65) | 7.66 (7.61; 7.72) | 7.82 (7.77; 7.88) | 0.0004 |  |  |
|  | Model 2^3^ | 7.81 (7.75; 7.86) | 7.61 (7.56; 7.66) | 7.58 (7.53; 7.63) | 7.67 (7.61; 7.72) | 7.89 (7.84; 7.95) | 0.98 | 0.00 (-0.02; 0.02) | <0.0001 |

DSR: dietary species richness; Q: quintile; GHG: greenhouse gases; PNNS: Programme National Nutrition Santé

^1^ species accounting for less than 5% (in grams) of any of the 33 food groups to which they pertain, were not accounted for in the total, plant or animal DSR computations, respectively.

^2^ Model 1 is adjusted for alcohol-free energy intake.

^3^ Model 2 is Model 1 further adjusted for age and sex.

^4^ P-trend for linear contrasts across quintiles.

^5^ P-value for linear association.

Table S5: Sustainability indicators and overall DSR (cut-off 5%)^1^ assessed as quintiles or as a continuous, model adjusted for age, sex, alcohol-free energy intake and the amount of plant protein in the diet

|  | Q1 (n=5758; 2-62) | Q2 (n=5759; 63-68) | Q3 (n=6418; 69-72) | Q4 (n=5620; 73-77) | Q5 (n=5618; 78-103) | P-trend^2^ | Increment /10 species | P-value^3^ |
| --- | --- | --- | --- | --- | --- | --- | --- | --- |
| **GHG emisssions (kg eqCO_2_ / day)** | | |  |  |  |  |  |  |
|  | 4.15 (4.10; 4.20) | 4.12 (4.07; 4.17) | 4.07 (4.03; 4.11) | 4.02 (3.97; 4.06) | 3.91 (3.86; 3.95) | <0.0001 | -0.09 (-0.07; -0.10) | <0.0001 |
| **Land occupation (m^2^ / day)** | | |  |  |  |  |  |  |
|  | 10.66 (10.52; 10.79) | 10.70 (10.57; 10.84) | 10.60 (10.48; 10.73) | 10.58 (10.45; 10.71) | 10.49 (10.35; 10.62) | 0.017 | -0.07 (-0.13; -0.01) | <0.0001 |
| **Energy demand (MJ / day)** | | |  |  |  |  |  |  |
|  | 18.33 (18.21; 18.45) | 17,81 (17,69; 17,93) | 17.62 (17.50; 17.73) | 17.41 (17.29; 17.53) | 16.96 (16.84; 17.08) | <0.0001 | -0.49 (-0.54; -0.43) | <0.0001 |
| **pRecipe** | |  |  |  |  |  |  |  |
|  | 0.70 (0.70; 0.71) | 0.70 (0.70; 0.71) | 0.70 (0.69; 0.70) | 0.69 (0.68; 0.70) | 0.68 (0.67;0.69) | 0.0001 | -0.01 (-0.01; 0.00) | <0.0001 |
| **comprehensive Dietary Quality Index (cDQI)** | | | |  |  |  |  |  |
|  | 49.91 (49.70; 50.12) | 50.67 (50.46; 50.88) | 51.33 (51.13; 51.53) | 52.07 (51.87; 52.28) | 53.56 (53.35; 53.78) | <0.0001 | 1.19 (1.10; 1.28) | <0.0001 |
| **Simplified PNNS Guidelines score - 2nd version (sPNNS-GS2)** | | | | |  |  |  |  |
|  | 2.49 (2.42; 2.55) | 2.56 (2.49; 2.63) | 2.62 (2.56; 2.68) | 2.83 (2.76; 2.90) | 3.15 (3.08; 3.22) | <0.0001 | 0.21 (0.18; 0.24) | <0.0001 |
| **Share of organic food of the diet (%)** | | |  |  |  |  |  |  |
|  | 0.24 (0.23; 0.24) | 0.26 (0.25; 0.26) | 0.28 (0.27; 0.28) | 0.32 (0.31; 0.33) | 0.39 (0.39; 0.40) | <0.0001 | 0.05 (0.05; 0.05) | <0.0001 |
| **Food Groups-based Dietary Diversity Score (FG-DDS)** | | | |  |  |  |  |  |
|  | 8.45 (8.40; 8.50) | 8.79 (8.74; 8.83) | 8.98 (8.93; 9.02) | 9.18 (9.13; 9.23) | 9.46 (9.41; 9.51) | <0.0001 | 0.36 (0.34; 0.38) | <0.0001 |
| **Monetary cost of the diet (€ / day)** | | |  |  |  |  |  |  |
|  | 7.83 (7.78; 7.88) | 7.65 (7.60; 7.70) | 7.62 (7.57; 7.67) | 7.66 (7.61; 7.71) | 7.79 (7.73;7.84) | 0.0009 | -0.04 (-0.06; -0.02) | 0.0001 |

DSR: dietary species richness; Q: quintile; GHG: greenhouse gases; PNNS: Programme National Nutrition Santé.

^1^ species accounting for less than 5% (in grams) of any of the 33 food groups to which they pertain, are not accounted for in the DSR computation.

^2^ P-trend for linear contrasts across quintiles.

^3^ P-value for linear association.

Table S6: Sustainability indicators and plant DSR (cut-off 5%)^1^ assessed by quintiles or as a continuous variable

|  | Q1 (n=6120; 2;40) | Q2 (n=5900; 41–45) | Q3 (n=5917; 46–49) | Q4 (n=5216; 50–54) | Q5 (n=6020; 55–78) | P-trend^4^ | Increment / 10 species | P-value^5^ |
| --- | --- | --- | --- | --- | --- | --- | --- | --- |
| **GHG emissions (kg eqCO_2_ / day)** | | |  |  |  |  |  |  |
| Model 4^2^ | 4.58 (4.53; 4.63) | 4.30 (4.25; 4.35) | 4.08 (4.03; 4.13) | 3.87 (3.82; 3.92) | 3.40 (3.35; 3.45) | <0.0001 | -0.50 (-1.04; -0.48) | <0.0001 |
| Model 5^3^ | 4.40 (4.35; 4.45) | 4.21 (4.16; 4.26) | 4.05 (4.00; 4.10) | 3.94 (3.89; 4.00) | 3.65 (3.60; 3.70) | <0.0001 | -0.33 (-0.36; -0.30) | <0.0001 |
| **Land occupation (m^2^ / day)** | |  |  |  |  |  |  |  |
| Model 4^2^ | 11.70 (11.56; 11.84) | 11.15 (11.01; 11.29) | 10.61 (10.47; 10.75) | 10.24 (10.09; 10.39) | 9.28 (9.14; 9.42) | <0.0001 | -1.04 (-1.12; -0.97) | <0.0001 |
| Model 5^3^ | 11.53 (11.39; 11.68) | 11.06 (10.92; 11.20) | 10.59 ( 10.45; 10.73) | 10.30 ( 10.15; 10.45) | 9.50 ( 9.35; 9.64) | <0.0001 | -0.89 (-0.97; -0.81) | <0.0001 |
| **Energy demand (MJ / day)** | |  |  |  |  |  |  |  |
| Model 4^2^ | 19.09 (18.96; 19.22) | 18.24 (18.11; 18.37) | 17.77 (17.64; 17.90) | 17.15 (17.01; 17.29) | 15.82 (15.69; 15.95) | <0.0001 | -1.41 (-1.48; -1.34) | <0.0001 |
| Model 5^3^ | 18.54 (18.41; 18.67) | 17.97 (17.84; 18.09) | 17.67 (17.55; 17.80) | 17.36 (17.23; 17.50) | 16.56 (16.43; 16.69) | <0.0001 | -0.88 (-0.95; -0.81) | <0.0001 |
| **pRecipe** |  |  |  |  |  |  |  |  |
| Model 4^2^ | 0.77 (0.76; 0.78) | 0.73 (0.72; 0.74) | 0.70 (0.69; 0.71) | 0.67 (0.66; 0.68) | 0.60 (0.59 ; 0.61) | <0.0001 | -0.07 (-0.07; -0.06) | <0.0001 |
| Model 5^3^ | 0.75 (0.75; 0.76) | 0.72 (0.72; 0.73) | 0.69 (0.69; 0.70) | 0.68 (0.67; 0.69) | 0.63 (0.62; 0.63) | <0.0001 | -0.06 (-0.06 ; -0.05) | <0.0001 |
| **comprehensive Dietary Quality Index (cDQI)** | | |  |  |  |  |  |  |
| Model 4^2^ | 48.25 (48.04; 48.46) | 50.35 (50.14; 50.56) | 51.53 (51.32; 51.74) | 52.70 (52.48; 52.93) | 54.82 (54.61; 55.03) | <0.0001 | 2.75 (2.64; 2.86) | <0.0001 |
| Model 5^3^ | 49.21 (49.00; 49.42) | 50.83 (50.63; 51.04) | 51.69 (51.48; 51.89) | 52.33 (52.12; 52.55) | 53.54 (53.34; 53.75) | <0.0001 | 1.82 (1.71; 1.94) | <0.0001 |
| **Simplified PNNS Guidelines Score – 2^nd^ version (sPNNS-GS2)** | | | |  |  |  |  |  |
| Model 4^2^ | 1.88 (1.81; 1.95) | 2.32 (2.24; 2.39) | 2.66 (2.59; 2.74) | 3.00 (2.93; 3.08) | 3.80 (3.73; 3.87) | <0.0001 | 0.80 (0.76; 0.84) | <0.0001 |
| Model 5^3^ | 2.20 (2.13; 2.27) | 2.48 (2.41; 2.55) | 2.72 (2.65; 2.79) | 2.88 (2.81; 2.95) | 3.37 (3.30; 3.44) | <0.0001 | 0.49 (0.45; 0.53) | <0.0001 |
| **Share of organic food in the diet (g/day, %)** | | |  |  |  |  |  |  |
| Model 4^2^ | 0.18 (0.18; 0.19) | 0.24 (0.23; 0.25) | 0.28 (0.27; 0.28) | 0.34 (0.33; 0.35) | 0.45 (0.44; 0.45) | <0.0001 | 0.11 (0.11; 0.11) | <0.0001 |
| **Food Groups-based Dietary Diversity Score (FG-DDS)** | | | |  |  |  |  |  |
| Model 4^2^ | 8.36 (8.32; 8.41) | 8.76 (8.71; 8.81) | 8.97 (8.93; 9.02) | 9.21 (9.16; 9.26) | 9.56 (9.52; 9.61) | <0.0001 | 0.59 (0.49; 0.54) | <0.0001 |
| Model 5^3^ | 8.46 (8.41; 8.51) | 8.81 (8.76; 8.86) | 8.99 (8.94; 9.04) | 9.18 (9.12; 9.23) | 9.43 (9.39; 9.48) | <0.0001 | 0.42 (0.40; 0.45) | <0.0001 |
| **Monetary cost of the diet (€ / day)** | | |  |  |  |  |  |  |
| Model 4^2^ | 7.51 (7.46; 7.56) | 7.56 (7.51; 7.61) | 7.67 (7.62; 7.72) | 7.77 (7.72; 7.83) | 8.03 (7.98; 8.08) | <0.0001 | 0.22 (0.19; 0.25) | <0.0001 |
| Model 5^3^ | 7.76 (7.71; 7.81) | 7.68 (7.63; 7.73) | 7.71 (7.66; 7.76) | 7.68 (7.63; 7.73) | 7.71 (7.65; 7.76) | 0.11 | -0.02 (-0.05; 0.01) | 0.11 |

DSR: dietary species richness; Q: quintile; GHG: greenhouse gases; PNNS: Programme National Nutrition Santé

^1^ species accounting for less than 5% (in grams) of any of the 33 food groups to which they pertain, were not accounted for in the total, plant or animal DSR computations, respectively.

^2^ Model 4 is adjusted for age, sex, alcohol-free energy intake, and animal DSR (cut-off 5%, continuous).

^3^Model 5 is Model 4 further adjusted for the share of organic food in the diet.

^4^P-trend for linear contrasts across quintiles.

^5^P-value for linear association.

Table S7: Sustainability indicators and animal DSR (cut-off 5%)^1^ assessed by quintiles or as a continuous variable

|  | Q1 (n=5875; 0-19) | Q2 (n=5081; 20-21) | Q3 (n=7332; 22-23) | Q4 (n=5743; 24-25) | Q5 (n=5142; 26-33) | P-trend^4^ | Increment / 10 species | P-value^5^ |
| --- | --- | --- | --- | --- | --- | --- | --- | --- |
| **GHG emissions (kg eqCO_2_ / day)** | | |  |  |  |  |  |  |
| Model 6^2^ | 3.38 (3.33; 3.43) | 4.15 (4.10; 4.21) | 4.22 (4.17; 4.26) | 4.27 (4.22; 4.32) | 4.25 (4.19; 4.30) | <0.0001 | 1.02 (0.96; 1.08) | <0.0001 |
| Model 7^3^ | 3.52 (3.47; 3.57) | 4.11 (4.06; 4.17) | 4.16 (4.12; 4.21) | 4.23 (4.18; 4.28) | 4.25 (4.20; 4.30) | <0.0001 | 0.84 (2.16; 2.49) | <0.0001 |
| **Land occupation (m^2^ / day)** | |  |  |  |  |  |  |  |
| Model 6^2^ | 8.99 (8.85; 9.13) | 10.77 (10.62; 10.92) | 10.94 (10.82; 11.07) | 11.15 (11.01; 11.30) | 11.21 (11.05; 11.36) | <0.0001 | 2.48 (2.32; 2.64) | <0.0001 |
| Model 7^3^ | 9.12 (8.97; 9.26) | 10.73 (10.58; 10.88) | 10.89 (10.76; 11.02) | 11.12 (10.98; 11.26) | 11.21 (11.06; 11.36) | <0.0001 | 2.33 (2.16; 2.49) | <0.0001 |
| **Energy demand (MJ / day)** | |  |  |  |  |  |  |  |
| Model 6^2^ | 16.40 (16.27; 16.54) | 17.96 (17.82; 18.10) | 18.04 (17.92; 18.15) | 17.96 (17.82; 18.09) | 17.76 (17.62; 17.90) | <0.0001 | 1.95 (1.81; 2.10) | <0.0001 |
| Model 7^3^ | 16.81 (16.69; 16.94) | 17.85 (17.71; 17.98) | 17.87 (17.76; 17.98) | 17.84 (17.71; 17.97) | 17.77 (17.63; 17.90) | <0.0001 | 1.40 (1.26; 1.55) | <0.0001 |
| **pRecipe** |  |  |  |  |  |  |  |  |
| Model 6^2^ | 0.59 (0.58; 0.60) | 0.71 (0.70; 0.72) | 0.72 (0.71; 0.73) | 0.73 (0.72; 0.74) | 0.73 (0.72; 0.74) | <0.0001 | 0.16 (0.15; 0.17) | <0.0001 |
| Model 7^3^ | 0.60 (0.59; 0.61) | 0.70 (0.70; 0.71) | 0.71 (0.71; 0.72) | 0.73 (0.72; 0.74) | 0.73 (0.72; 0.74) | <0.0001 | 0.14 (0.13; 0.15) | <0.0001 |
| **comprehensive Dietary Quality Index (cDQI)** | | | |  |  |  |  |  |
| Model 6^2^ | 54.17 (53.96; 54.38) | 51.66 (51.43; 51.89) | 50.68 (50.49; 50.87) | 50.48 (50.27; 50.70) | 50.57 (50.34; 50.79) | <0.0001 | -3.15 (-3.39; -2.91) | <0.0001 |
| Model 7^3^ | 53.48 (53.27; 53.69) | 51.85 (51.63; 52.07) | 50.96 (50.77; 51.14) | 50.68 (50.47; 50.88) | 50.55 (50.34; 50.77) | <0.0001 | -2.19 (-2.43; -1.96) | <0.0001 |
| **Simplified PNNS Guidelines Score – 2^nd^ version (sPNNS-GS2)** | | | |  |  |  |  |  |
| Model 6^2^ | 3.62 (3.55; 3.70) | 2.65 (2.57; 2.73) | 2.41 (2.34; 2.47) | 2.47 (2.39; 2.54) | 2.51 (2.43; 2.59) | <0.0001 | -1.28 (-1.36; -1.20) | <0.0001 |
| Model 7^3^ | 3.38 (3.31; 3.45) | 2.72 (2.64; 2.79) | 2.51 (2.44; 2.57) | 2.54 (2.46; 2.61) | 2.50 (2.43; 2.58) | <0.0001 | -0.96 (-1.04; -0.88) | <0.0001 |
| **Share of organic food in the diet (%)** | | |  |  |  |  |  |  |
| Model 6^2^ | 0.38 (0.37; 0.39) | 0.27 (0.27; 0.28) | 0.26 (0.26; 0.27) | 0.27 (0.27; 0.28) | 0.30 (0.29; 0.30) | <0.0001 | -0.11 (-0.12; -0.11) | <0.0001 |
| **Food Groups-based Dietary Diversity Score (FG-DDS)** | | | |  |  |  |  |  |
| Model 6^2^ | 9.08 (9.03 ; 9.12) | 8.95 (8.90; 9.00) | 8.93 (8.89 ; 8.98) | 8.97 (8.92; 9.02) | 8.90 (8.85; 8.95) | 0.18 | -0.03 (-0.09; -0.02) | 0.18 |
| Model 7^3^ | 9.01 (8.96; 9.06) | 8.97 (8.92; 9.02) | 8.96 (8.92; 9.02) | 8.99 (8.94; 9.04) | 8.90 (8.85; 8.95) | 0.05 | 0.06 (0.00; 0.11) | 0.05 |
| **Monetary cost of the diet (€ / day)** | | |  |  |  |  |  |  |
| Model 6^2^ | 8.26 (8.21; 8.31) | 7.68 (7.63; 7.74) | 7.57 (7.52; 7.61) | 7.52 (7.47; 7.57) | 7.51 (7.46; 7.57) | <0.0001 | -0.84 (-0.90; -0.78) | <0.0001 |
| Model 7^3^ | 8.07 (8.02; 8.12) | 7.73 (7.68; 7.79) | 7.64 (7.60; 7.69) | 7.57 (7.52; 7.62) | 7.51 (7.45; 7.56) | <0.0001 | -0.59 (-0.65; -0.53) | <0.0001 |

DSR: dietary species richness; Q: quintile; GHG: greenhouse gases; PNNS: Programme National Nutrition Santé

^1^ species accounting for less than 5% (in grams) of any of the 33 food groups to which they pertain, were not accounted for in the total, plant or animal DSR computations, respectively.

^2^Model 6 is adjusted for age, sex, alcohol-free energy intake, and plant DSR (cut-off 5%, continuous).

^3^Model 7 is Model 5 further adjusted on the share of organic food in the diet.

^4^P-trend for linear contrasts across quintiles.

^5^P-value for linear association.

Table S8: Characteristics of the study sample across total DSR quintiles (cut-off 5g)^1^

|  | Q1 (n=5728) | Q2 (n=5531) | Q3 (n=6224) | Q4 (n=5976) | Q5 (n=5714) | P-trend^3^ |
| --- | --- | --- | --- | --- | --- | --- |
| Total DSR | < 34 | 35-41 | 42-48 | 49-56 | > 57 |  |
| **Female, %** | 74.96 | 73.99 | 75.48 | 74.82 | 74.64 | 0.88 |
| **Male, %** | 25.04 | 26.01 | 24.52 | 25.18 | 25.36 |  |
| **Age, years, mean (SD)** | 49.0 (15.1) | 52.4 (14.4) | 54.0 (13.8) | 55.4 (13.1) | 56.8 (12.2) | <0.0001 |
| **Occupational status, %** |  |  |  |  |  |  |
| Unemployed | 9.40 | 8.21 | 9.01 | 8.65 | 9.80 | <0.0001 |
| Student | 3.43 | 1.94 | 1.47 | 1.72 | 1.13 |  |
| Retired | 26.98 | 35.17 | 38.30 | 41.23 | 44.48 |  |
| Farmer. Self-employed | 1.91 | 1.80 | 1.67 | 1.71 | 1.66 |  |
| Intellectual profession | 22.20 | 22.65 | 21.50 | 19.98 | 18.72 |  |
| Employee | 18.26 | 14.31 | 12.67 | 11.41 | 10.31 |  |
| Manual worker | 1.11 | 1.12 | 1.22 | 0.63 | 0.67 |  |
| Intermediate profession | 16.70 | 14.81 | 14.15 | 14.69 | 13.24 |  |
| **Educational level, %** |  |  |  |  |  |  |
| < High school diploma | 21.54 | 21.66 | 20.76 | 20.68 | 21.85 | 0.15 |
| High school diploma | 15.76 | 14.61 | 14.86 | 14.06 | 14.04 |  |
| > High school diploma | 62.70 | 63.74 | 64.39 | 65.26 | 64.10 |  |
| **Monthly income per household unit (€), %** | | |  |  |  |  |
| <1200 | 14.63 | 11.82 | 10.59 | 10.50 | 10.31 | <0.0001 |
| 1200-2300 | 42.74 | 39.40 | 39.43 | 37.96 | 37.40 |  |
| 2300-3700 | 25.4 | 30.22 | 30.12 | 31.97 | 31.12 |  |
| > 3700 | 10.25 | 13.11 | 13.82 | 13.99 | 14.73 |  |
| Unwilling to answer | 6.83 | 5.46 | 6.03 | 5.58 | 6.44 |  |
| **Smoking status, %** |  |  |  |  |  |  |
| Non smoker | 49.81 | 49.13 | 48.93 | 48.21 | 47.90 | <0.0001 |
| Former smoker | 35.29 | 39.05 | 41.37 | 42.32 | 44.22 |  |
| Current smoker | 14.90 | 11.81 | 9.70 | 9.47 | 7.88 |  |
| **Physical activity level (MET-min/week), %** | | |  |  |  |  |
| Missing data^2^ | 11.85 | 11.15 | 10.28 | 11.11 | 9.47 | <0.0001 |
| High (> 1500) | 25.51 | 31.18 | 32.52 | 36.52 | 42.10 |  |
| Moderate (600-1500) | 35.34 | 35.76 | 38.62 | 36.54 | 35.81 |  |
| Low (<600) | 27.31 | 21.91 | 18.59 | 15.83 | 12.62 |  |
| **Alcohol-free energy intake, mean (SD)** | |  |  |  |  |  |
|  | 1488 (472) | 1734 (484) | 1914 (493) | 2105 (526) | 2455 (608) | <0.0001 |

DSR: dietary species richness; Q: quintile; SD: standard deviation. MET: metabolic equivalent of task.

^1^ species accounting for less than 5g are not accounted for in the DSR computation.

^2^ Missing data as some questions are optional.

^3^ P-values refer to two-sided tests for linear contrasts across quintiles for continuous variables or the Mantel-Haenszel Chi-square trend for categorical variables.

Table S9: Sustainability indicators and total DSR (cut-off 5g)^1^ assessed by quintiles or as a continuous variable

|  |  | |  | Q1 (n=5728, <34) | Q2 (n=5531, 35-41) | Q3 (n=6224, 42-48) | Q4 (n=5976, 49-56) | Q5 (n=5714, >57) | P-trend^4^ | Increment / 10 species | P-value^5^ |
| --- | --- | --- | --- | --- | --- | --- | --- | --- | --- | --- | --- |
|  | | **GHG emissions (kg eqCO_2_ / day)** | | | |  |  |  |  |  |  |
|  |  | | Model 1^2^ | 4.50 (4.44; 4.55) | 4.34 (4.29; 4.39) | 4.17 (4.11; 4.22) | 3.90 (3.85; 3.95) | 3.37 (3.31; 3.43) | <0.0001 |  |  |
|  |  | | Model 2^3^ | 4.54 (4.48; 4.59) | 4.34 (4.29; 4.39) | 4.16 (4.11; 4.22) | 3.88 (3.83; 3.94) | 3.35 (3.30; 3.41) | <0.0001 | -0.36 (-0.38; -0.34) | <0.0001 |
|  | | **Land occupation (m^2^ / day)** | | |  |  |  |  |  |  |  |
|  |  | | Model 1^2^ | 11.50 (11.34; 11.66) | 11.21 (11.07; 11.35) | 10.84 (10.69; 10.99) | 10.28 (10.14; 10.42) | 9.23 (9.07; 9.38) | <0.0001 |  |  |
|  |  | | Model 2^3^ | 11.62 (11.46; 11.78) | 11.21 (11.07; 11.35) | 10.83 (10.68; 10.97) | 10.23 (10.09; 10.37) | 9.17 (9.01; 9.33) | <0.0001 | -0.75 (-0.81; -0.69) | <0.0001 |
|  | | **Energy demand (MJ / day)** | | |  |  |  |  |  |  |  |
|  |  | | Model 1^2^ | 17.04 (16.89; 17.19) | 17.66 (17.53; 17.79) | 17.85 (17.71; 17.99) | 17.86 (17.73; 18.00) | 17.72 (17.57; 17.87) | <0.0001 |  |  |
|  |  | | Model 2^3^ | 17.22 (17.07; 17.37) | 17.69 (17.56; 17.82) | 17.83 (17.69; 17.97) | 17.79 (17.65; 17.92) | 17.61 (17.46; 17.76) | <0.0001 | 0.09 (0.03; 0.15) | <0.0001 |
|  | | **pRecipe** | |  |  |  |  |  |  |  |  |
|  |  | | Model 1^2^ | 0.75 (0.74; 0.76) | 0.74 (0.73; 0.74) | 0.71 (0.70; 0.72) | 0.68 (0.67; 0.68) | 0.60 (0.59; 0.61) | <0.0001 |  |  |
|  |  | | Model 2^3^ | 0.76 (0.75; 0.77) | 0.74 (0.73; 0.74) | 0.71 (0.70; 0.72) | 0.67 (0.66; 0.68) | 0.60 (0.59; 0.61) | <0.0001 | -0.05 (-0.05; -0.05) | <0.0001 |
|  | | **comprehensive Dietary Quality Index (cDQI)** | | | |  |  |  |  |  |  |
|  |  | | Model 1^2^ | 43.37 (43.15; 43.60) | 48.53 (48.33; 48.73) | 51.69 (51.48; 51.91) | 54.55 (54.35; 54.75) | 59.11 (58.88; 59.33) | <0.0001 |  |  |
|  |  | | Model 2^3^ | 44.30 (44.08; 44.51) | 48.87 (48.67; 49.06) | 51.64 (51.43; 51.84) | 54.18 (53.99; 54.38) | 58.29 (58.07; 58.50) | <0.0001 | 4.09 (4.01; 4.18) | <0.0001 |
|  | | **Simplified PNNS Guidelines score 2 (sPNNS-GS2)** | | | | |  |  |  |  |  |
|  |  | | Model 1^2^ | 0.58 (0.50; 0.66) | 1.92 (1.85; 1.98) | 2.78 (2.71; 2.85) | 3.60 (3.53; 3.67) | 4.68 (4.61; 4.76) | <0.0001 |  |  |
|  |  | | Model 2^3^ | 0.65 (0.57; 0.73) | 1.95 (1.89; 2.02) | 2.78 (2.71; 2.85) | 3.58 (3.51; 3.65) | 4.60 (4.53; 4.68) | <0.0001 | 1.14 (1.11; 1.17) | <0.0001 |
|  | | **Share of organic food on the diet (%)** | | | |  |  |  |  |  |  |
|  |  | | Model 1^2^ | 0.14 (0.13; 0.15) | 0.22 (0.22; 0.23) | 0.28 (0.27; 0.29) | 0.35 (0.34; 0.35) | 0.48 (0.47; 0.49) | <0.0001 |  |  |
|  |  | | Model 2^3^ | 0.14 (0.13; 0.15) | 0.22 (0.22; 0.23) | 0.28 (0.27; 0.29) | 0.35 (0.34; 0.36) | 0.48 (0.47; 0.49) | <0.0001 | 0.10 (0.10; 0.10) | <0.0001 |
|  | | **Food Groups-based Dietary Diversity Score (FG-DDS)** | | | | |  |  |  |  |  |
|  |  | | Model 1^2^ | 6.69 (6.65; 6.73) | 8.26 (8.22; 8.29) | 9.09 (9.05; 9.14) | 9.87 (9.83; 9.91) | 10.86 (10.82; 10.90) | <0.0001 |  |  |
|  |  | | Model 2^3^ | 6.77 (6.72; 6.81) | 8.28 (8.24; 8.32) | 9.09 (9.05; 9.13) | 9.84 (9.80; 9.88) | 10.80 (10.76; 10.85) | <0.0001 | 1.15 (1.13; 1.16) | <0.0001 |
|  | | **Monetary cost of the diet (€ / day)** | | | |  |  |  |  |  |  |
|  |  | | Model 1^2^ | 6.56 (6.51; 6.62) | 7.14 (7.09; 7.19) | 7.51 (7.46; 7.56) | 8.01 (7.96; 8.06) | 9.29 (9.23; 9.34) | <0.0001 |  |  |
|  |  | | Model 2^3^ | 6.65 (6.60; 6.71) | 7.16 (7.11; 7.20) | 7.50 (7.45; 7.55) | 7.97 (7.92; 8.02) | 9.23 (9.18; 9.28) | <0.0001 | 0.79 (0.77; 0.81) | <0.0001 |

DSR: dietary species richness; Q: quintile; GHG: greenhouse gases; PNNS: Programme National Nutrition Santé.

^1^ species accounting for less than 5g are not accounted for in the DSR computation.

^2^ Model 1 is adjusted for alcohol-free energy intake.

^3^ Model 2 is Model 1 further adjusted for age and sex.

^4^P-trend for linear contrasts across quintiles.

^5^P-value for linear association.

Table S10: Sustainability indicators and total DSR (cut-off 5g)^1^ assessed by quintiles or as a continuous variable, model adjusted for age, sex, alcohol-free energy intake and plant protein in the diet

|  | Q1 (n=5728, <34) | Q2 (n=5531, 35-41) | Q3 (n=6224, 42-48) | Q4 (n=5976, 49-56) | Q5 (n=5714, >57) | P-trend^2^ | Increment / 10 species | P-value^3^ |
| --- | --- | --- | --- | --- | --- | --- | --- | --- |
| **GHG emissions (kg eqCO2 / day)** | | |  |  |  |  |  |  |
|  | 4.05 (4.00; 4.11) | 4.03 (3.99; 4.08) | 4.06 (4.01; 4.11) | 4.03 (3.99; 4.08) | 4.09 (4.04; 4.15) | 0.17 | 0.02 (-0.01; 0.03) | 0.17 |
| **Land occupation (m^2^ / day)** | | |  |  |  |  |  |  |
|  | 10.51 (10.36; 10.66) | 10.49 (10.36; 10.62) | 10.58 (10.45; 10.72) | 10.57 (10.44; 10.70) | 10.89 (10.73; 11.04) | 0.0001 | 0.12 (0.06; 0.19) | 0.0001 |
| **Energy demand (MJ / day)** | | |  |  |  |  |  |  |
|  | 15.93 (15.79; 16.06) | 16.85 (16.74; 16.97) | 17.55 (17.43; 17.67) | 18.18 (18.06; 18.30) | 19.59 (19.46; 19.73) | <0.0001 | 1.14 (1.08; 1.19) | <0.0001 |
| **pRecipe** | |  |  |  |  |  |  |  |
|  | 0.69 (0.68 ; 0.70) | 0.69 (0.68 ; 0.70) | 0.69 (0.69; 0.70) | 0.69 (0.69; 0.70) | 0.71 (0.71; 0.72) | <0.0001 | 0.01 (0.01; 0.01) | <0.0001 |
| **comprehensive Dietary Quality Index (cDQI)** | | | |  |  |  |  |  |
|  | 45.18 (44.96; 45.40) | 49.44 (49.25; 49.63) | 51.83 (51.63; 52.03) | 53.91 (53.72; 54.10) | 56.92 (56.70; 57.14) | <0.0001 | 3.52 (3.43; 3.61) | <0.0001 |
| **Simplified PNNS Guidelines score - 2nd version (sPNNS-GS2)** | | | | |  |  |  | |
|  | 1.24 (1.17; 1.32) | 2.33 (2.27; 2.40) | 2.91 (2.84; 2.97) | 3.40 (3.33; 3.46) | 3.70 (3.62; 3.77) | <0.0001 | 0.71 (0.67; 0.74) | <0.0001 |
| **Share of organic food in the diet, %** | | |  |  |  |  |  |  |
|  | 0.18 (0.17; 0.19) | 0.25 (0.25; 0.26) | 0.29 (0.28; 0.30) | 0.34 (0.33; 0.34) | 0.42 (0.41; 0.42) | <0.0001 | 0.07 (0.07; 0.07) | <0.0001 |
| **Food Groups-based Dietary Diversity Score (FG-DDS)** | | | |  |  |  |  |  |
|  | 6.72 (6.68; 6.77) | 8.25 (8.21; 8.29) | 9.08 (9.04; 9.12) | 9.85 (9.81; 9.89) | 10.87 (10.82; 10.91) | <0.0001 | 1.22 (1.20; 1.24) | <0.0001 |
| **Monetary cost of the diet (€ / day)** | | |  |  |  |  |  |  |
|  | 6.76 (6.71; 6.82) | 7.23 (7.18; 7.27) | 7.53 (7.47; 7.58) | 7.94 (7.89; 7.99) | 9.06 (9.01; 9.12) | <0.0001 | 0.73 (0.70; 0.75) | <0.0001 |

DSR: dietary species richness; Q: quintile; GHG: greenhouse gases; PNNS: Programme National Nutrition Santé.

^1^ species accounting for less than 5g are not accounted for in the DSR computation.

^2^ P-trend for linear contrasts across quintiles.

^3^ P-value for linear association

Table S11: Sustainability indicators and plant DSR (cut-off 5g)^1^ assessed by quintiles or as a continuous variable

|  | Q1 (n=6075; <28) | Q2 (n=5381;29-34) | Q3 (n=5949;34-40) | Q4 (n=5817;41-47) | Q5 (n=5951; >48) | P-trend^4^ | Increment / 10 species | P-value^5^ |
| --- | --- | --- | --- | --- | --- | --- | --- | --- |
| **GHG emissions (kg eqCO_2_ / day)** | | |  |  |  |  |  |  |
| Model S4^2^ | 4.72 (4.67; 4.77) | 4.39 (4.35; 4.44) | 4.16 (4.11; 4.21) | 3.83 (3.78; 3.88) | 3.14 (3.08; 3.19) | <0.0001 | -0.53 (-0.55; -0.51) | <0.0001 |
| Model S5^3^ | 4.47 (4.42; 4.52) | 4.27 (4.23; 4.32) | 4.13 (4.08; 4.19) | 3.92 (3.87; 3.97) | 3.46 (3.40; 3.51) | <0.0001 | -0.36 (-0.38; -0.33) | <0.0001 |
| **Land occupation (m^2^ / day)** | | |  |  |  |  |  |  |
| Model S4^2^ | 12.02 (11.87; 12.17) | 11.30 (11.16; 11.44) | 10.82 (10.67; 10.97) | 10.12 (9.99; 10.26) | 8.73 (8.57; 8.88) | <0.0001 | -1.11 (-1.17; -1.04) | <0.0001 |
| Model S5^3^ | 11.81 (11.65; 11.96) | 11.20 (11.06; 11.34) | 10.79 (10.65; 10.94) | 10.19 (10.06; 10.33) | 9.00 (8.84; 9.16) | <0.0001 | -0.99 (-1.06; -0.91) | <0.0001 |
| **Energy demand (MJ / day)** | |  |  |  |  |  |  |  |
| Model S4^2^ | 18.19 (18.06; 18.32) | 18.05 (17.93; 18.17) | 17.85 (17.72; 17.98) | 17.47 (17.35; 17.59) | 16.55 (16.42; 16.69) | <0.0001 | -0.58 (-0.64; -0.52) | <0.0001 |
| Model S5^3^ | 17.31 (17.18; 17.44) | 17.62 (17.50; 17.74) | 17.75 (17.63; 17.87) | 17.78 (17.67; 17.89) | 17.69 (17.56; 17.83) | <0.0001 | 0.08 (0.02; 0.14) | <0.0001 |
| **pRecipe** |  |  |  |  |  |  |  |  |
| Model S4^2^ | 0.79 (0.78; 0.80) | 0.74 (0.73; 0.75) | 0.71 (0.70; 0.72) | 0.66 (0.66; 0.67) | 0.57 (0.56; 0.58) | <0.0001 | -0.07 (-0.08; -0.07) | <0.0001 |
| Model S5^3^ | 0.77 (0.76; 0.78) | 0.73 (0.72; 0.74) | 0.71 (0.70; 0.72) | 0.67 (0.66; 0.68) | 0.60 (0.59; 0.61) | <0.0001 | -0.06 (-0.06; -0.06) | <0.0001 |
| **comprehensive Dietary Quality Index (cDQI)** | | |  |  |  |  |  |  |
| Model S4^2^ | 45.33 (45.12; 45.54) | 49.20 (49.01; 49.40) | 51.47 (51.27; 51.68) | 53.93 (53.74; 54.12) | 57.68 (57.47; 57.90) | <0.0001 | 3.99 (3.90; 4.08) | <0.0001 |
| Model S5^3^ | 46.41 (46.20; 46.62) | 49.72 (49.53; 49.92) | 51.59 (51.39; 51.80) | 53.55 (53.37; 53.74) | 56.30 (56.08; 56.52) | <0.0001 | 3.29 (3.19; 3.39) | <0.0001 |
| **Simplified PNNS Guidelines score - 2nd version (sPNNS-GS2)** | | | |  |  |  |  |  |
| Model S4^2^ | 0.57 (0.50; 0.64) | 1.86 (1.79; 1.92) | 2.72 (2.65; 2.80) | 3.65 (3.59; 3.72) | 4.87 (4.79; 4.94) | <0.0001 | 1.38 (1.35; 1.41) | <0.0001 |
| Model S5^3^ | 0.88 (0.81; 0.95) | 2.01 (1.94; 2.08) | 2.76 (2.69; 2.83) | 3.54 (3.48; 3.61) | 4.47 (4.39; 4.54) | <0.0001 | 1.18 (1.15; 1.22) | <0.0001 |
| **Share of organic food in the diet, %** | | |  |  |  |  |  |  |
| Model S4^2^ | 0.14 (0.13; 0.15) | 0.22 (0.21; 0.23) | 0.28 (0.27; 0.29) | 0.35 (0.34; 0.36) | 0.49 (0.49; 0.50) | <0.0001 | 0.12 (0.11; 0.12) | <0.0001 |
| **Food Groups-based Dietary Diversity Score (FG-DDS)** | | | |  |  |  |  |  |
| Model S4^2^ | 7.13 (7.08; 7.17) | 8.41 (8.37; 8.45) | 9.03 (8.99; 9.07) | 9.75 (9.71; 9.79) | 10.55 (10.51; 10.59) | <0.0001 | 1.08 (1.06; 1.10) | <0.0001 |
| Model S5^3^ | 7.13 (7.08; 7.17) | 8.41 (8.37; 8.45) | 9.03 (8.99; 9.07) | 9.75 (9.71; 9.79) | 10.55 (10.51; 10.60) | <0.0001 | 1.11 (1.09; 1.13) | <0.0001 |
| **Monetary cost of the diet (€ / day)** | | |  |  |  |  |  |  |
| Model S4^2^ | 6.94 (6.89; 6.99) | 7.24 (7.19; 7.29) | 7.51 (7.46; 7.56) | 7.91 (7.86; 7.95) | 8.98 (8.93; 9.03) | <0.0001 | 0.70 (0.68; 0.72) | <0.0001 |
| Model S5^3^ | 7.21 (7.16; 7.26) | 7.38 (7.33; 7.42) | 7.54 (7.50; 7.59) | 7.81 (7.77; 7.86) | 8.63 (8.57; 8.68) | <0.0001 | 0.52 (0.49; 0.54) | <0.0001 |

DSR: dietary species richness; Q: quintile; GHG: greenhouse gases; PNNS: Programme National Nutrition Santé.

^1^ species accounting for less than 5g are not accounted for in the DSR computation.

^2^ Model S4 is adjusted for age, sex, alcohol-free energy intake and animal DSR (cut-off 5g, continuous).

^3^ Model S5 is Model S4 further adjusted for the share of organic food in the diet.

^4^ P-trend for linear contrasts across quintiles.

^5^ P-value for linear association.
Table S12: Sustainability indicators and animal DSR (cut-off 5g)^1^ assessed by quintiles or as a continuous variable

|  | Q1 (n=4994; <4) | Q2 (n=4493; 5-5) | Q3 (n=9007; 6-7) | Q4 (n=6127; 8-9) | Q5 (n=4552; >10) | P-trend^4^ | Increment / 10 species | P-value^5^ |
| --- | --- | --- | --- | --- | --- | --- | --- | --- |
| **GHG emissions (kg eqCO_2_ / day)** | | |  |  |  |  |  |  |
| Model S6^2^ | 3.33 (3.27; 3.38) | 3.87 (3.81; 3.93) | 4.11 (4.06; 4.15) | 4.29 (4.23; 4.35) | 4.64 (4.59; 4.69) | <0.0001 | 1.68 (1.59; 1.77) | <0.0001 |
| Model S7^3^ | 3.37 (3.32; 3.42) | 3.88 (3.83; 3.94) | 4.10 (4.06; 4.14) | 4.27 (4.21; 4.33) | 4.62 (4.57; 4.66) | <0.0001 | 1.58 (1.49; 1.67) | <0.0001 |
| **Land occupation (m^2^ / day)** | |  |  |  |  |  |  |  |
| Model S6^2^ | 8.93 (8.79; 9.08) | 10.29 (10.13; 10.45) | 10.78 (10.65; 10.90) | 11.13 (10.96; 11.29) | 11.84 (11.70; 11.98) | <0.0001 | 3.54 (3.29; 3.78) | <0.0001 |
| Model S7^3^ | 8.97 (8.82; 9.11) | 10.30 (10.14; 10.46) | 10.77 (10.65; 10.89) | 11.11 (10.95; 11.28) | 11.82 (11.69; 11.96) | <0.0001 | 3.47 (3.21; 3.71) | <0.0001 |
| **Energy demand (MJ / day)** | |  |  |  |  |  |  |  |
| Model S6^2^ | 14.76 (14.63; 14.88) | 16.33 (16.19; 16.48) | 17.46 (17.35; 17.57) | 18.61 (18.46; 18.76) | 20.80 (20.67; 20.92) | <0.0001 | 8.64 (8.42; 8.86) | <0.0001 |
| Model S7^3^ | 14.94 (14.81; 15.06) | 16.38 (16.24; 16.51) | 17.43 (17.33; 17.53) | 18.52 (18.38; 18.66) | 20.69 (20.57; 20.81) | <0.0001 | 8.26 (8.05; 8.46) | <0.0001 |
| **pRecipe** |  |  |  |  |  |  |  |  |
| Model S6^2^ | 0.58 (0.57; 0.59) | 0.67 (0.66; 0.68) | 0.71 (0.70; 0.71) | 0.73 (0.72; 0.74) | 0.79 (0.78; 0.79) | <0.0001 | 0.25 (0.24; 0.27) | <0.0001 |
| Model S7^3^ | 0.59 (0.58; 0.59) | 0.67 (0.66; 0.68) | 0.70 (0.70; 0.71) | 0.73 (0.72; 0.74) | 0.78 (0.77; 0.79) | <0.0001 | 0.25 (0.23; 0.26) | <0.0001 |
| **comprehensive Dietary Quality Index (cDQI)** | | |  |  |  |  |  |  |
| Model S6^2^ | 49.05 (48.85; 49.25) | 50.15 (49.94; 50.37) | 51.55 (51.38; 51.71) | 52.54 (52.31; 52.77) | 53.98 (53.79; 54.18) | <0.0001 | 6.42 (6.08; 6.76) | <0.0001 |
| Model S7^3^ | 48.86 (48.67; 49.06) | 50.11 (49.90; 50.32) | 51.58 (51.41; 51.74) | 52.63 (52.41; 52.86) | 54.09 (53.90; 54.28) | <0.0001 | 6.83 (6.49; 7.17) | <0.0001 |
| **Simplified PNNS Guidelines Score, 2nd version (sPNNS-GS2)** | | | |  |  |  |  |  |
| Model S6^2^ | 3.09 (3.02; 3.16) | 3.03 (2.96; 3.11) | 3.00 (2.94; 3.06) | 2.48 (2.40; 2.56) | 1.99 (1.92; 2.05) | <0.0001 | -1.47 (-1.59; -1.35) | <0.0001 |
| Model S7^3^ | 3.04 (2.97; 3.11) | 3.02 (2.95; 3.09) | 3.01 (2.95; 3.06) | 2.51 (2.43; 2.59) | 2.02 (1.95; 2.08) | <0.0001 | -1.35 (-1.47; -1.24) | <0.0001 |
| **Share of organic food in the diet, %** | | |  |  |  |  |  |  |
| Model S6^2^ | 0.33 (0.32; 0.33) | 0.30 (0.30; 0.31) | 0.29 (0.29; 0.30) | 0.28 (0.27; 0.29) | 0.28 (0.27; 0.28) | <0.0001 | -0.07 (-0.08; -0.06) | <0.0001 |
| **Food Groups-based Dietary Diversity Score (FG-DDS)** | | | |  |  |  |  |  |
| Model S6^2^ | 7.89 (7.85; 7.93) | 8.49 (8.44; 8.53) | 9.07 (9.04; 9.11) | 9.53 (9.48; 9.57) | 9.82 (9.78; 9.86) | <0.0001 | 2.30 (2.23;2.36) | <0.0001 |
| Model S7^3^ | 7.90 (7.86; 7.94 | 8.49 (8.45; 8.53) | 9.07 (9.04; 9.10) | 9.52 (9.48; 9.57) | 9.81 (9.78; 9.85) | <0.0001 | 2.28 (2.21; 2.35) | <0.0001 |
| **Monetary cost of the diet (€ / day)** | | |  |  |  |  |  |  |
| Model S6^2^ | 7.32 (7.27; 7.37) | 7.33 (7.28; 7.39) | 7.56 (7.52; 7.60) | 7.80 (7.74; 7.85) | 8.46 (8.42; 8.51) | <0.0001 | 1.78 (1.70; 1.87) | <0.0001 |
| Model S7^3^ | 7.27 (7.22; 7.32) | 7.32 (7.27; 7.38) | 7.57 (7.53; 7.61) | 7.82 (7.76; 7.88) | 8.49 (8.44; 8.54) | <0.0001 | 1.89 (1.81; 1.97) | <0.0001 |

DSR: dietary species richness; Q: quintile; GHG: greenhouse gases; PNNS: Programme National Nutrition Santé.

^2^ Model S6 is adjusted for age, sex, alcohol-free energy intake and plant DSR (cut-off 5g, continuous).

^3^ Model S7 is Model S6 further adjusted for the share of organic food in the diet.

^4^ P-trend for linear contrasts across quintiles.

^5^ P-value for linear association.

Figure S1: Sustainability indicators and plant DSR (cut-off 5%)^1^ assessed by quintiles or as a continuous variable, further adjusted for the share of organic food in the diet^2^

Figure S2: Sustainability indicators and animal DSR (cut-off 5%)^1^ assessed by quintiles or as a continuous variable, further adjusted for the share of organic food in the diet^2^
